# Plasma polymerized bio-interface directs fibronectin adsorption and functionalization to enhance “epithelial barrier structure” formation via FN-ITG β1-FAK-mTOR signaling cascade

**DOI:** 10.1186/s40824-022-00323-0

**Published:** 2022-12-26

**Authors:** Shoucheng Chen, Zhuwei Huang, Rahul Madathiparambil Visalakshan, Haiwen Liu, Akash Bachhuka, You Wu, Panthihage Ruvini L. Dabare, Pu Luo, Runheng Liu, Zhuohong Gong, Yin Xiao, Krasimir Vasilev, Zhuofan Chen, Zetao Chen

**Affiliations:** 1 Hospital of Stomatology, Guanghua School of Stomatology, Sun Yat-Sen University and Guangdong Provincial Key Laboratory of Stomatology, No.56, Lingyuan West Road, Yuexiu District, Guangzhou, 510055 China; 2 Oregon Health & Science University, Portland, USA; 3 Department of Electronics, Electric and Automatic Engineering, Rovira i Virgili University (URV), Tarragona, 43003 Spain; 4 Academic Unit of Science, Technology, Engineering and Mathematics (STEM), University of South Australia, Mawson Lakes, SA 5095 Australia; 5 Institute of Health and Biomedical Innovation, Queensland University of Technology, Brisbane, 4059 Australia

**Keywords:** Transepithelial medical devices, Plasma polymerization, Epithelial barrier structure, Protein adsorption

## Abstract

**Background:**

Transepithelial medical devices are increasing utilized in clinical practices. However, the damage of continuous natural epithelial barrier has become a major risk factor for the failure of epithelium-penetrating implants. How to increase the “epithelial barrier structures” (focal adhesions, hemidesmosomes, etc*.*) becomes one key research aim in overcoming this difficulty. Directly targeting the in situ “epithelial barrier structures” related proteins (such as fibronectin) absorption and functionalization can be a promising way to enhance interface-epithelial integration.

**Methods:**

Herein, we fabricated three plasma polymerized bio-interfaces possessing controllable surface chemistry. Their capacity to adsorb and functionalize fibronectin (FN) from serum protein was compared by Liquid Chromatography-Tandem Mass Spectrometry. The underlying mechanisms were revealed by molecular dynamics simulation. The response of gingival epithelial cells regarding the formation of epithelial barrier structures was tested.

**Results:**

Plasma polymerized surfaces successfully directed distinguished protein adsorption profiles from serum protein pool, in which plasma polymerized allylamine (ppAA) surface favored adsorbing adhesion related proteins and could promote FN absorption and functionalization via electrostatic interactions and hydrogen bonds, thus subsequently activating the ITG β1-FAK-mTOR signaling and promoting gingival epithelial cells adhesion.

**Conclusion:**

This study offers an effective perspective to overcome the current dilemma of the inferior interface-epithelial integration by in situ protein absorption and functionalization, which may advance the development of functional transepithelial biointerfaces.

**Graphical Abstract:**

Tuning the surface chemistry by plasma polymerization can control the adsorption of fibronectin and functionalize it by exposing functional protein domains. The functionalized fibronectin can bind to human gingival epithelial cell membrane integrins to activate epithelial barrier structure related signaling pathway, which eventually enhances the formation of epithelial barrier structure.

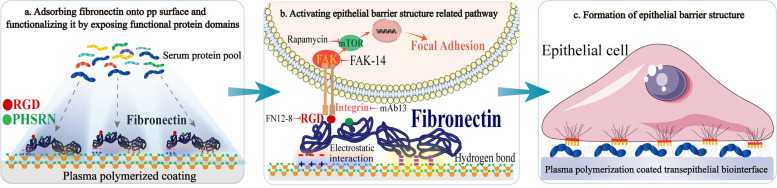

**Supplementary Information:**

The online version contains supplementary material available at 10.1186/s40824-022-00323-0.

## Introduction

**Fig. 1:**
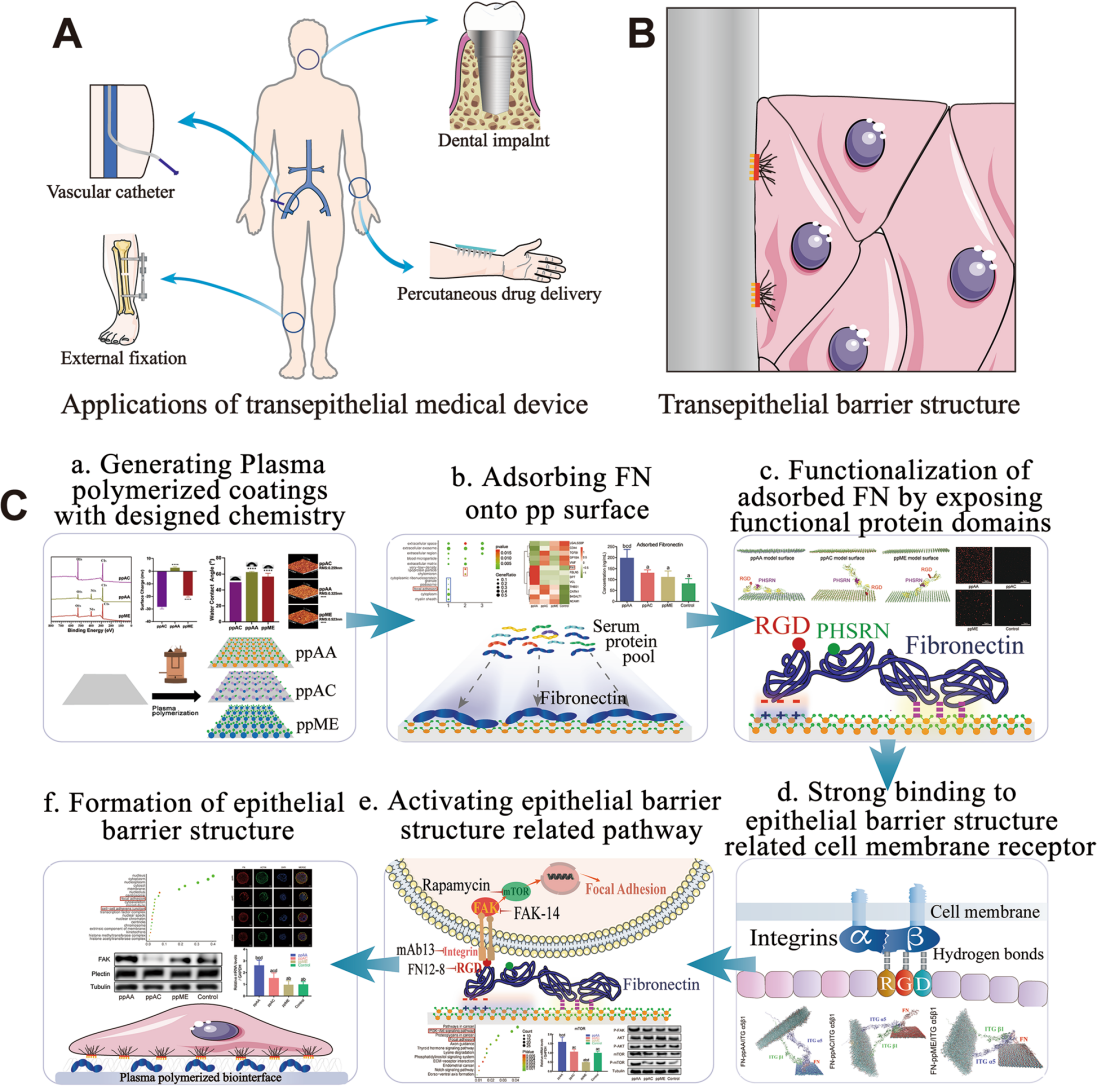
**A** Transepithelial medical devices are widely used in clinical practice and the clinical demand of transepithelial integration is very high. **B** The transepithelial integration is mainly determined by the amount epithelial barrier structures, which occur at the interface-epithelium interface. **C** Experimental flow of the study. a) Surface coatings with designed chemistry were generated by plasma polymerization. b) Serum protein absorption profile on each surface was dissected by Liquid Chromatography-Tandem Mass Spectrometry and the adsorption amount of key protein, fibronectin, was detected by ELISA assay. c) The conformational change and exposal of functional protein domains of adsorbed fibronectin were detected by Molecular Dynamics Simulation and verified by immunofluorescence (IF). d) The activation of human gingival epithelial cell membrane integrins by adsorbed fibronectin was analyzed by molecular docking calculation, real-time quantitative polymerase chain reaction (RT-qPCR), Western blot (WB) and IF. e) The activation of epithelial barrier structure related pathway was investigated using RNA-sequencing (RNA-seq), RT-qPCR and WB. f) The formation of epithelial barrier structure and cell adhesion behavior were eventually evaluated using RNA-seq, RT-qPCR, WB, Structured Illumination Microscopy, scanning electron microscope (SEM) and cell shedding test

Transepithelial medical devices have drawn increasing interests for the purpose to improve the convenience, efficiency and comfort of treatment [1, 2]. Transepithelial osseointegrated prosthetics (limbs, ears, dental implants, etc*.*) significantly improve the comfort and quality for tissue repair [3, 4]. Transepithelial drug delivery devices such as indwelling catheters and microneedles offer easy-access, minimal-invasive and high-efficacy treatment approaches [5] (Fig. 1A). While transepithelial devices have apparent advantages, one disadvantage the damage of continuous natural epithelial barrier has limited their applications, especially when hard metal implants need to integrate with soft epithelial tissue. It remains difficult to rebuild the integrated epithelial barrier on the transepithelial interfaces [6].

The epithelium tends to down-migrate along the implant interface rather than establishing firm epithelial seal, resulting in poor resistance to mechanic avulsion and stimulus from external environment, thus has become the major risk factor for infection and even the failure of epithelium-penetrating implants [7]. It has been reported that 38% to 56% of transepithelial devices are infected within five years after implantation [8]. Efforts are urgently needed to improve the integration between the epithelium and bio-interface.

Different with natural epithelial barrier, transepithelial interface-epithelial barrier (Fig. 1B) is sustained by the formation of structures compromising focal adhesions, hemidesmosomes, etc [9, 10]. These “epithelial barrier structures” are highly potent biochemical complexes, which are established by functionalized extracellular adhesion-related proteins to contact cell membrane integrin receptors, which in turn activate the assembly of intracellular complexes [11]. How to increase these “epithelial barrier structures” becomes the key regulatory target in mimicking the natural integrated epithelial barrier [12].

To achieve this aim, some studies have tried to coat the bio-interfaces directly with extracellular adhesion-related proteins, including fibronectin (FN), laminin (LN), and collagen types I and IV (CI and CIV), etc [13, 14]. Among them, functionalized FN was considered one of the most efficient proteins to enhance the formation of “epithelial barrier structures” [15, 16]. However, when the modified surfaces encounter blood or tissue fluid, in situ protein adsorption is instantly and inevitably initiated on material surface through physicochemical interactions, leading to the possible conformational dysfunction and interaction blockade of the original coated protein [17, 18]. It is also of high cost to apply exogenous protein coating, which restricts the applications [8]. Meanwhile, the supply of FN on the bio-interface is adequate by taking into account the prime in situ interaction between the bio-interface and abundant autologous proteins [19]. Rather than focusing on the utilization of exogenous FN, it might be more feasible to absorb and functionalize autologous FN from in situ protein pool.

Modifying the surface chemistry with chemical groups (-NH_2_, -COOH, -OH, etc*.*) by self-assembled monolayers has been showed to have the capacity to induce the distinguished adsorption behaviors of FN on the surface [20]. This indicates chemical group modification could be a possible way of absorption and functionalization of FN from in situ protein pool, thus inducing “epithelial barrier structures”. However, the requirements of the pre-modification and limited substrate selection of such approaches restrict their practical applications on the various transepithelial medical devices [21].

Previous studies have introduced plasma polymerization as a feasible technique to accurately and controllably regulate the chemical composition, hydrophobicity, surface charge, etc*.* of biomaterials surfaces [22]. Meanwhile, our previous study showed that the polymerized coatings own unique advantages of similar crosslinked structure to natural peptides, which gives its strong and stable interaction capacity with adsorbed proteins [23]. In addition, plasma polymerization requires neither special substrate nor pre-modification, therefore can generate specific coatings on diverse mature medical devices [24]. Moreover, studies suggest that after proper optimization, plasma polymerized coatings show excellent retentivity and durability that can withstand ultrasonic bath [25], shaking bath [26] and even autoclaving process [27]. These features suggest that plasma polymerization could be an appealing method for the fabrication of designed chemical surfaces that can modulate FN absorption and functionalization from in situ protein pool.

In the study, we fabricated three plasma polymerized bio-interfaces possessing controllable surface chemistry and uniform chemistry-independent properties. The distinguished protein adsorption modulation capacities from serum protein pool were validated, and the detailed biological properties of these protein adsorption profiles were dissected. The FN adsorption and functionalization properties of the surfaces were evaluated. In addition, the continuous protein mediated signal transduction process and their effect on regulating “epithelial barrier structures” formation and epithelial cell integration were unveiled (Fig. 1C).

## Results

### Preparation of plasma polymerized interfaces with designed chemistry

In the study, we used allylamine (AA), acrylic acid (AC), and 2-methyl-oxazoline (ME) as the three precursors (Figure S1A) for plasma polymerization (pp) with the controlled deposition parameters to modify the tissue culture plate (TCP) surface into three distinct chemical surfaces, namely ppAA, ppAC, ppME, which were chemically rich in -NH_2_, -COOH, -CH_3_ groups, respectively. TCP without plasma polymer modification was used as the control surface.

**Fig. 2:**
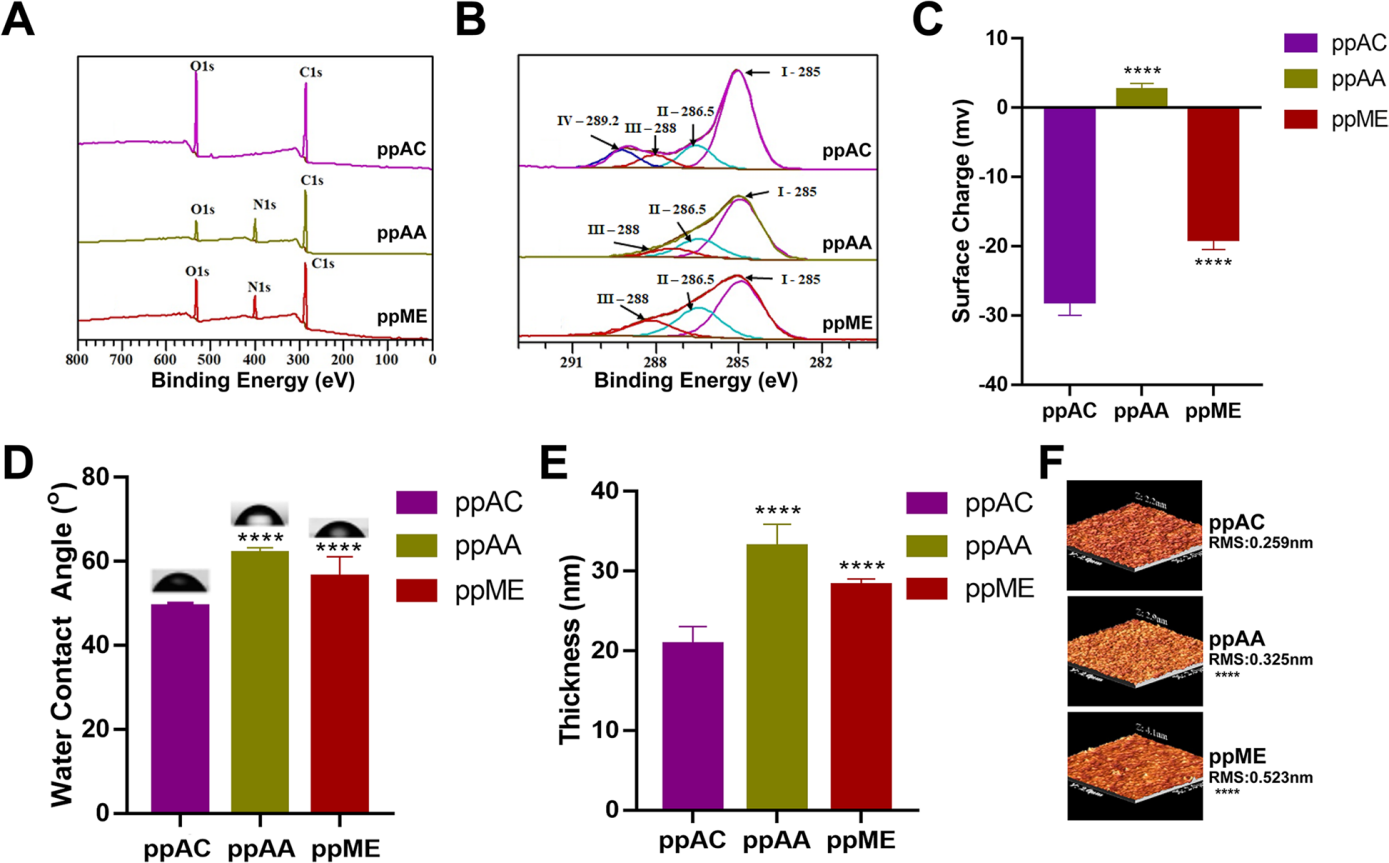
Physicochemical characterization of the allylamine (ppAA), acrylic acid (ppAC), and methyl-oxazoline (ppME) plasma polymerization modified surfaces. **A** Survey spectra from the XPS analysis. **B** C1s spectra; **C** Surface charge; **D** Static water contact angle; **E** Ellipsometry data showing the thickness of plasma deposition layers of the three modified chemical surfaces; **F** AFM images showing the surface topography and Root Mean Square Roughness (RMS) of the modified surfaces. *: *p* < 0.05, **: *p* < 0.01, ***: *p* < 0.001, , ****: *p* < 0.0001

Characterization of the three pp surfaces showed that their chemical compositions were in accordance with their designed chemistry. Specifically, XPS survey spectra (Fig. 2A) showed they presented chemical elements that were consistent with their original precursor molecules, that is, carbon (C1s peak) and oxygen (O1s peak) in ppAC, carbon, oxygen, and nitrogen (N1s peak) in ppAA, and carbon, oxygen, and nitrogen in ppME [28]. The deconvolution of the C1s peaks (Fig. 2B) showed that C1s of ppAC comprised of aliphatic carbon (-CH) at 285.0 eV, carbon bonded to single oxygen (C-OR) at 286.5 eV, carbon double bonded to oxygen (C = O) at 288 eV and carbon bonded to two oxygen atoms such as in acid/ester groups (COOR) at 289.2 eV. Whereas, C1s of both ppAA and ppME can accommodate three components: aliphatic carbon (-CH) at 285 eV, carbon bonded to single nitrogen (-C-NH_2_, C-NH-C and C-N = C) at 286.5 eV and carbon bonded to tertiary amines (imine: C = N or nitrile: C≡N) at 288 eV [29].

The surface charge of the three surfaces ranged from -28 mV to + 2.5 mV (Fig. 2C), which were in accordance with the nature of their corresponding chemical groups and with previous reports [22, 30]. The water contact angles of the three chemical surfaces varied between 49° to 62° (Fig. 2D), indicating chemistry-derived differences in the surface wettability. Considering all three chemical coatings were thin, with thickness less than 33 nm (Fig. 2E), smooth, with RMS roughness values below 0.523 nm (Fig. 2F), and exhibited uniform surface morphology (Fig. 2F), the chemistry-independent properties were well controlled.

### Protein adsorption profile on plasma polymerized surfaces with different chemistry from serum

To study the protein absorption profiles on pp surfaces, we used fetal bovine serum, which is commonly used as a source to provide adsorbed proteins in vitro [31]. Surface chemistry tunes protein adsorption profile at three aspects: 1) adsorption selectivity of certain proteins, 2) amount of adsorbed proteins, and 3) conformational changes of adsorbed proteins. Liquid Chromatography-Tandem Mass Spectrometry (LC–MS/MS) experiments and bioinformatic analysis were firstly used to dissect the serum protein adsorption profile on plasma polymerized surfaces***.***

#### Protein adsorption selectivity on plasma polymerized surfaces with different chemistry

**Fig. 3:**
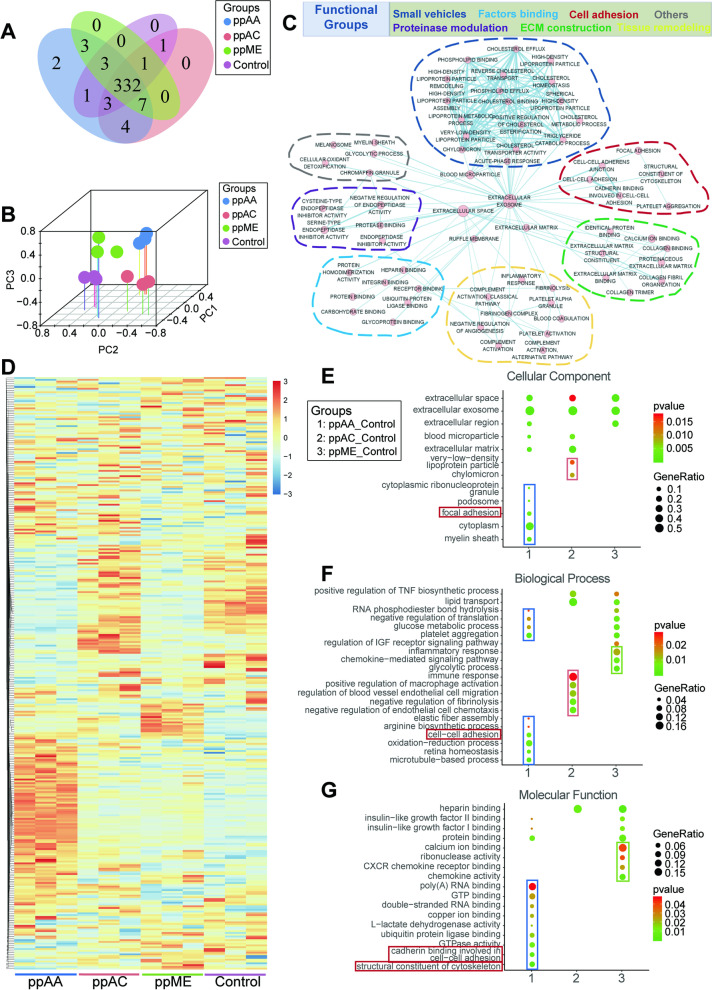
Bioinformatic analysis reveals the distinguished modulatory effect of adsorbed proteins on four surfaces. **A** Venn diagram of the number of protein types adsorbed on each surface; **B** Principal component analysis of co-adsorbed proteins of four surfaces; **C** Functional categorization of co-adsorbed proteins; **D** Heatmap analysis of co-adsorbed proteins; **E**–**G** Gene Ontology (GO) analysis of the upregulated proteins in the ppAA, ppAC, and ppME groups relative to the Control group. Cellular component (**E**), biological process (**F**), and molecular function (**G**)

Mass spectrometry results (iBAQ analysis) showed a constitutional adsorption similarity among each sample (Figure S2A), exhibiting wide-range functions (Figure S2B), thereby indicating the vast regulatory potentials of the adsorbed protein layer. The LFQ analysis results showed that 332 (over 92% of all the protein types detected) were co-adsorbed by all chemical surfaces (Fig. 3A), which can be divided into seven functional categories (*i.e.*, cell adhesion, ECM construction, small vehicles, tissue remodeling, factors binding, proteinase modulation and others) (Fig. 3C).

#### Amount of adsorbed serum proteins on plasma polymerized surfaces with different chemistry

Although most of the adsorbed protein types were co-adsorbed by all surfaces, their amount varied significantly among group (Fig. 3D), resulting in significant separation of samples from different groups during the principal component analysis (Fig. 3B). When compared to the control group, the ppAA, ppAC, and ppME groups adsorbed proteins of different functional characteristics. The ppAA group was mainly enriched with proteins related to the biological processes such as cell adhesion and metabolic regulation, ppAC with proteins responsible for immune regulation, while ppME with proteins involved in inflammation regulation and blood coagulation (Fig. 3E–G). The significantly differential adsorbed proteins between each plasma coating surfaces and TCP surface were showed in cluster heatmap in Figure S2C-E.

**Fig. 4:**
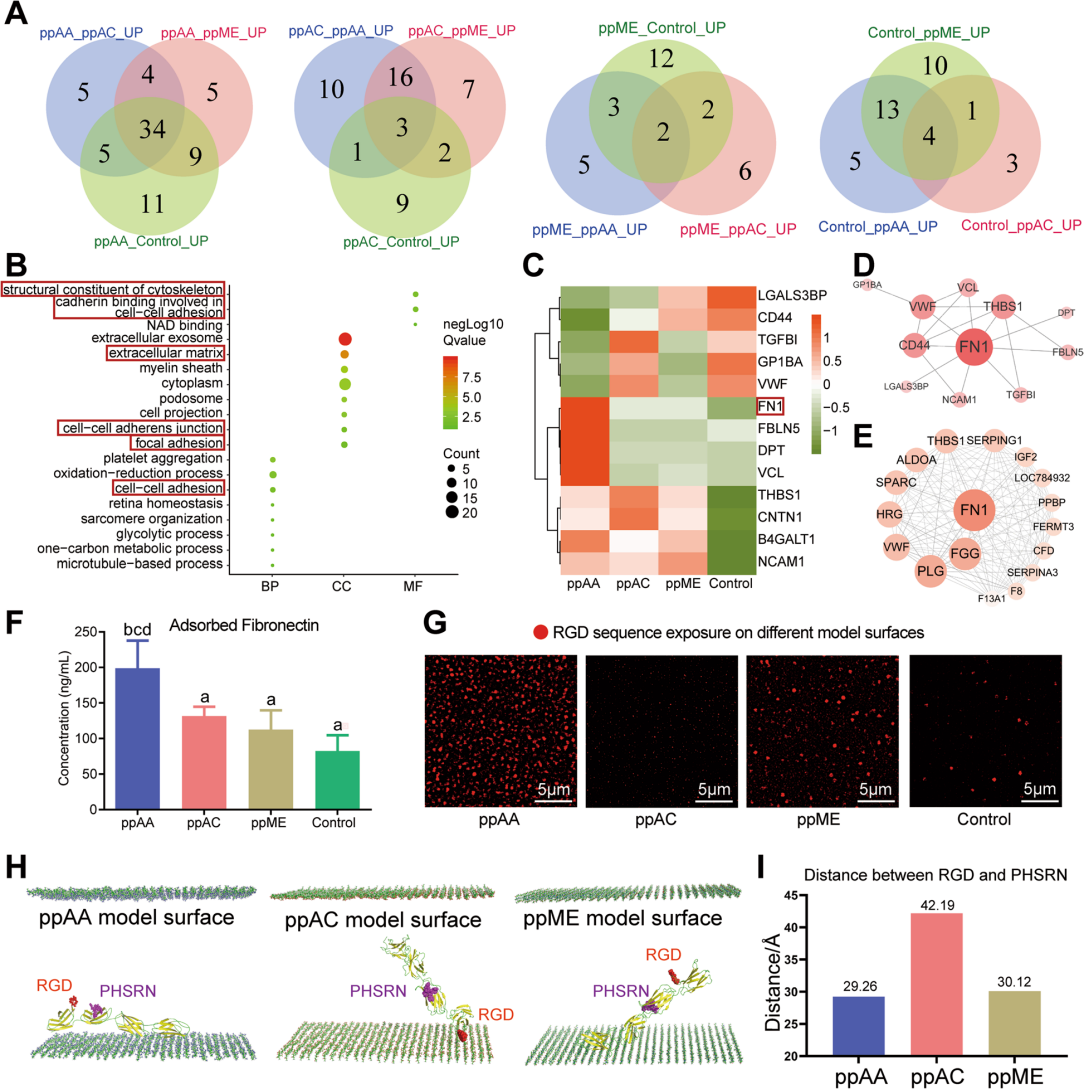
Functional analysis of the protein adsorption spectra and adsorption behavior evaluation of the representative adsorbed protein fibronectin (FN). **A** Number of upregulated proteins on each surface in comparison to the other surfaces. ppAA_ppAC_UP referred to the upregulated proteins in ppAA group compared with those in ppAC group, and the rest acronyms may be deduced by analogy; **B** GO enrichment analysis of the 34 upregulated proteins on the ppAA surface in comparison to all other surfaces on the biological process (BP), cellular process (CC) and molecular function (MF) level; **C**) Cluster heatmap of adhesion related adsorbed proteins on four surfaces; protein–protein interaction (PPI) analysis of cell adhesion related adsorbed proteins (**D**) and adsorbed proteins on ppAA surface (**E**); **F** ELISA of adsorbed FN on four surfaces. **G** Immunofluorescence of RGD sequence of FN exposure on four surfaces; **H** Molecular dynamics simulation showed stable conformation of adsorbed FN on different chemical surfaces at 60 ns, RGD (red) and PHSRN (purple); **I** Distance between the RGD and PHSRN in the stable conformation of FN on different chemical surfaces. Significant differences between two groups with a *p* < 0.05 are presented as follows: a) versus ppAA group, b) versus ppAC group, c) versus ppME group, and d) versus control group

Venn diagram of pairwise compared up-regulated proteins showed that there were 34 types of these upregulated adsorbed proteins were co-upregulated proteins in ppAA group compared to other three groups. While there were only 3, 2 and 4 co-upregulated proteins in ppAC, ppME and Control groups, respectively (Fig. 4A). Further GO enrichment analysis suggested that these 34 types co-upregulated adsorbed proteins of ppAA group were enriched in focal adhesion and extracellular matrix, and possess barrier structure formation related functions such as cell adhesion and cytoskeleton regulation (Fig. 4B). In addition, most upregulated adhesion related adsorbed proteins were observed in ppAA group (Fig. 4C).

#### Amount and conformational exposure of adsorbed FN on plasma polymerized surfaces with different chemistry

Further, the protein–protein interaction (PPI) analysis showed that fibronectin (FN) was at the key node of the PPI network of adhesion related adsorbed proteins (Fig. 4D), as well as the adsorbed proteins on ppAA group (Fig. 4E). This finding was supported by ELISA assay of the adsorbed FN in the four groups, in which we found that FN were adsorbed onto all four surfaces with the highest amount on ppAA (Fig. 4F). As the functions of a protein are significantly affected by its conformations [32], the conformational changes of fibronectin on the three plasma polymerized coating surfaces need further investigation. Immunofluorescence assay of RGD site showed the most of RGD sequence exposal on ppAA surface (Fig. 4G).

We further used molecular dynamics simulation (MDS) to investigate the adsorption kinetics and conformation change of FN adsorbed onto plasma polymerized surfaces at molecular and atomic scales [33].

The complex details of the protein-chemical surface interaction process were analyzed based on the system comprising structure information of the core fragment of the FN protein (FnIII_7-10_) (Figure S3A) on simulated chemical surface (Figure S3B), in the environment of a solvent (H_2_O) (Figure S3C). The systems reached an equilibrium state when the simulation reached 60 ns (Figure S3D). Owing to the distinct chemistries of the three plasma polymerized surfaces, they caused different degree of exposal of active epitope in the FN molecule, especially two synergetic cell-binding sites, RGD (Fig. 4H, red) and PHSRN (Fig. 4H, purple) [34]. The similar distance between the RGD and PHSRN (F ig. 4I) was found on ppAA and ppME surface, while increased distance was observed on ppAC surface.

### Interaction mechanisms of FN adsorbed onto plasma polymerized surfaces with different chemistry

**Fig. 5:**
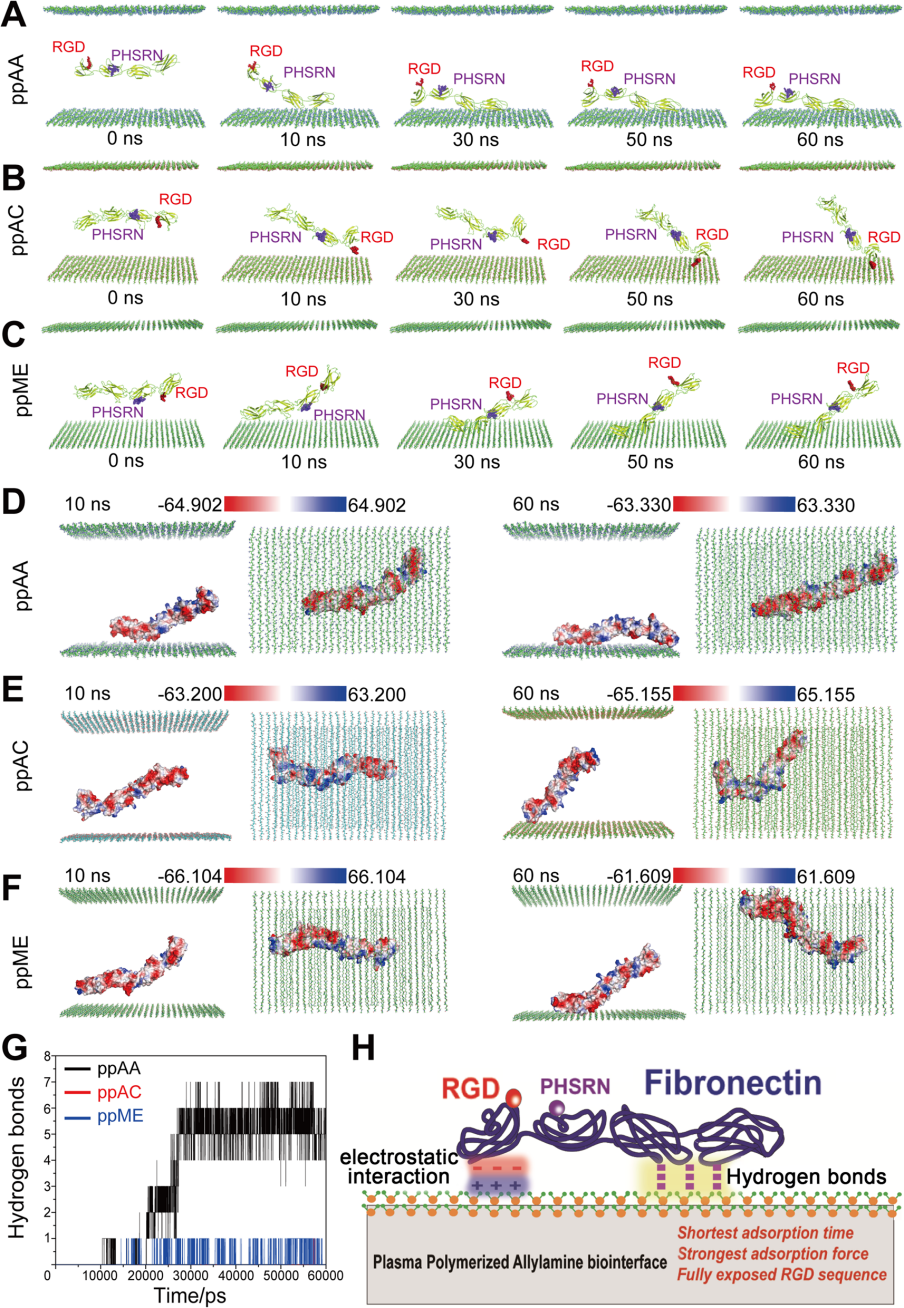
Molecular dynamic simulations of FnIII_7-10_ on ppAA, ppAC, and ppME surfaces. Adsorption process of FnIII_7-10_ on the ppAA surface (**A**), ppAC surface (**B**) and ppME surface (**C**), RGD (red) and PHSRN (purple); Formation of electrostatic interactions between FnIII_7-10_ and the ppAA surface (**D**), ppAC surface (**E**) and ppME surface (**F**) at 10 ns and 60 ns; **G** Numbers of hydrogen bonds between FnIII_7-10_ and each surface, and their alterations over time; **H** The illustration of how the physicochemical signals of ppAA surface translated into the protein signals of adsorbed fibronectin

On ppAA surface, the RGD and PHSRN sites were gradually exposed to the solvent during the process of MDS. When the protein conformation achieved stability, both of they were fully exposed to the solvent (Fig. 5A, Video 1), which shall favor the binding of the protein to the cell membrane [35]. On the ppAC surface, final equilibrium was achieved through conformation change in the protein chain structure with only PHSRN exposed to the solvent, while RGD was in contact with the ppAC surface in an up-side-down manner (Fig. 5B, Video 2). On the ppME surface, only RGD was exposed to solvent (Fig. 5C, Video 3), while the binding status between FN and ppME surface was unstable, indicating a weak compacity for it to guide cell adhesion. This can be explained by MDS from three aspects:

1. Shortest contact time of FN on ppAA. FN contacted with the ppAA surface within 10 ns and acquired stable adhesion within 30 ns through a progressive bending of protein conformation (Fig. 5A, Video 1). The contact time extended to 50 ns on the ppAC surface (Fig. 5B, Video 2). Although the first contact time on the ppME surface was 10 ns (Fig. 5C, Video 3), the binding stability was poor and the structural fluctuations persisted.
2. Strongest intermolecular forces between FN and ppAA. The FN adsorbed on ppAA surface owned the most hydrogen bonds (4 to 7) (Fig. 5G) and large-area electrostatic interaction (FnIII7, FnIII8, and FnIII10 negative-charge-enriching regions) with the surface at the equilibrium stage (Fig. 5D). During the simulation, the distance between the amino acid residues (ASP1263, ASP1221, ASP1222) that formed hydrogen bonding and the ppAA surface gradually decreased and was less than 0.2 nm after 30 ns (Figure S3F), further demonstrating the strong and stable hydrogen bonding between FN protein and ppAA surface. Although FN exhibited hydrogen bonds and electrostatic interactions with the ppME surface, it was significantly less than that on the ppAA surface (Fig. 5F and G), whereas the ppAC surface only exhibited electrostatic interactions (Fig. 5E) and no quantifiable hydrogen bonds (Fig. 5G).
3. High adsorption stability of FN on ppAA. The binding between FN and the ppAA surface do not dissociate once formed (Fig. 5A, Video 1). In contrast, the hydrogen bonds between FN and the ppME surface continued to break due to the presence of electrostatic repulsion during simulation, which was indicated by severe fluctuations in the solvent-accessible surface analysis (SASA, Figure S3E).

In addition, the secondary structure of FN in the ppAA, ppAC and ppME systems remained stable during the dynamic simulation process (Figure S4A). The FN conformations on the three surfaces at equilibrium stage were superimposed to its initial conformation (Figure S4B-D), which confirmed that the main structure of the protein remained unchanged and stable, ensuring the functional integrity of adsorbed FN on plasma polymerized coating surfaces.

### Further “epithelial barrier structures” formation from human gingival epithelial cells

Given that FN stood out from the protein adsorption profile on ppAA surface with good binding capacity and cell adhesion-favorable conformation (Fig. 5A & H), we assumed that the ppAA surface could improve epithelial cell adhesion, which is the biological fundamental of epithelial barrier structure. To verify this assumption, we used human gingival epithelial cells (HGEs) to test the cellular response to ppAA surface, including changes of cell membrane receptors, intercellular signaling pathway, and cell behavior. Before that, we first tested the cell cytotoxicity and apoptosis effect of the four plasma polymerized surfaces. CCK-8 assay showed that the first 6 h after seeding, the control group presented the lowest cytotoxicity, but after 24 h, HGEs in ppAA group showed the best viability, indicating its lowest cytotoxicity (Figure S1B). Flow cytometry showed that ppAA and ppAC group induced significantly less apoptotic cells than ppME and control group (Figure S1C). These results suggest the well in-vitro biocompatibility of ppAA surface.

#### Changes of cell membrane receptors

**Fig. 6:**
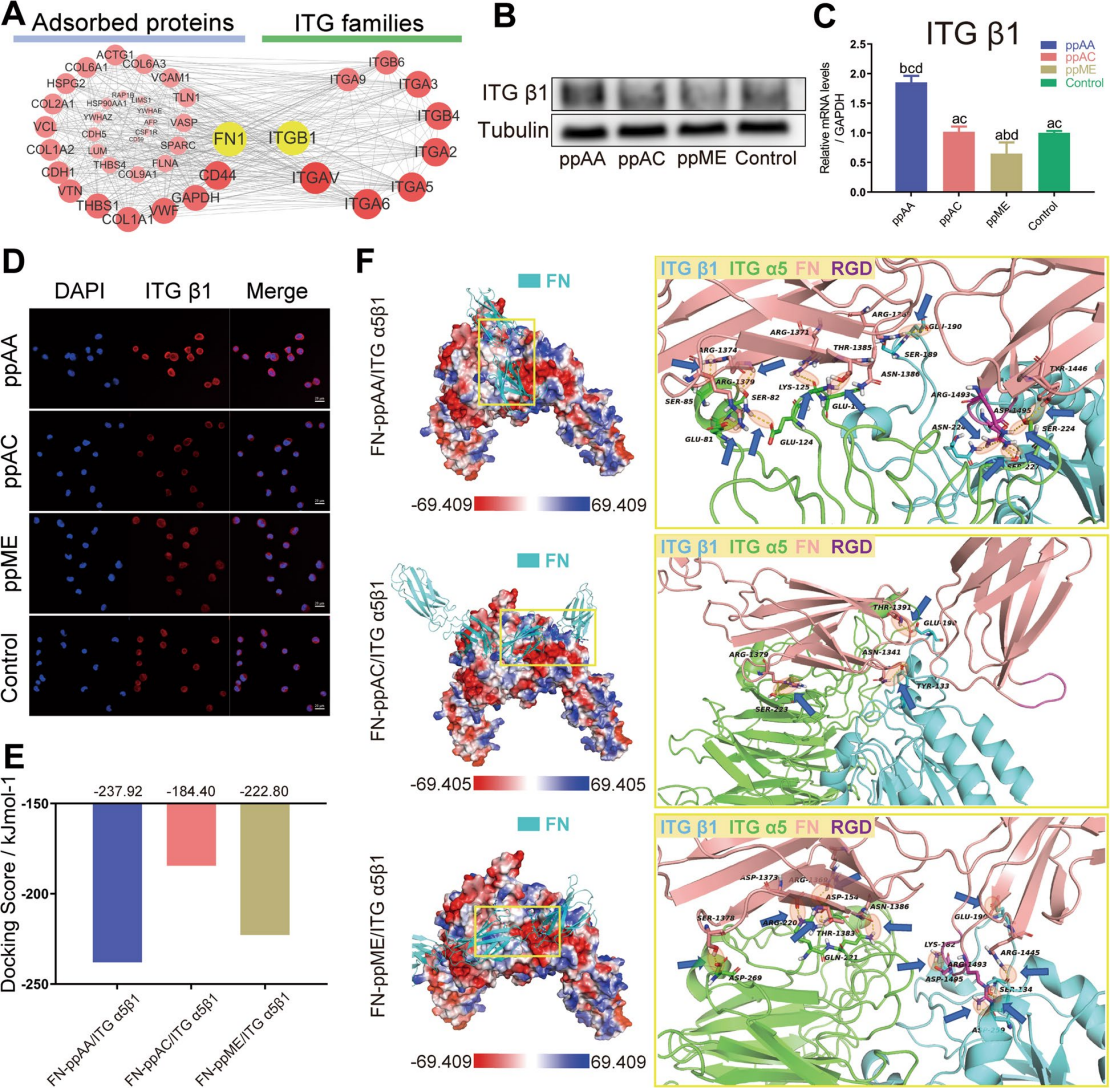
Changes in the membrane receptor of HGEs cultured on the chemical surfaces. **A** Interactions network between the adsorbed proteins and integrin (ITG) family; representative western blot images (**B**), RT-qPCR results (**C**) and immunofluorescence images (**D**) of the expression of ITG β1 of HGEs in four groups; **E** binding scores of the lowest energy binding conformations of each groups during molecular docking calculations of the FN/ITGα5β1-membrane complex; **F** the optimal binding conformation and the detailed hydrogen bonding interactions of the FN-ppAA/ITGα5β1-membrane complex (first row), FN-ppAC/ITGα5β1-membrane complex (second row) and FN-ppME/ITGα5β1-membrane complex (third row). Significant differences between two groups with a *p* < 0.05 are presented as follows: a) versus ppAA group, b) versus ppAC group, c) versus ppME group, and d) versus control group

FN mainly interacts with cells through its RGD site by specific binding to the integrin (ITG) superfamily of proteins [36]. The interactions network between the adsorbed proteins and integrin (ITG) family showed the most multiple connections between the adsorbed fibronectin and ITGβ1 (Fig. 6A), suggesting the ITGβ1 may play a major role in the responses to the adsorbed FN with distinct exposed RGD site. This was further confirmed by western blotting (WB, Fig. 6B), real-time quantitative PCR (RT-qPCR, Fig. 6C) and immunofluorescence (Fig. 6D), showing that HGEs in ppAA group had the highest expression of ITGβ1.

According to previous reports [37], residues 1491–1498 in the FN protein have the potential to interact with ITGα5β1. Thus, we performed molecular docking calculations to obtain the FN- chemical surfaces in complex with ITGα5β1so as to explore the effect of different FN conformations induced by the three surfaces on the binding ability of downstream ITGα5β1. The three-dimensional structure diagram of ITGα5β1 receptor protein on cell membrane was showed in Figure S4E. The molecular docking calculations were carried out to obtain the FN—chemical surfaces in complex with ITGα5β1 (Figure S4F). Docking results showed the lowest energy-binding-conformations of each groups, with binding scores showing that the ppAA group possessed the lowest (-237.92 kJ/mol) compared to ppAC (-184.40 kJ/mol) and ppME (-222.80 kJ/mol) group (Fig. 6E), indicating the optimal binding conformation of FN-ppAA surface has the best binding stability to ITGα5β1.

Further, the final binding conformation of the FN-ITGα5β1 complexes on different surfaces were illustrated (Fig. 6F). With the RGD site fully exposed to the solvent, FN on ppAA surface could directly insert into the binding site of ITGα5β1 through the RGD region to form a stable binding, while encountering significant steric hindrance on ppAC and ppME surface (Figure S4F). Moreover, the interaction of amino acid residues in the binding region showed that the RGD site of FN on ppAA surface have the most hydrogen bonds with ITGα5β1 protein (first row of Fig. 6F, 12 hydrogen bonds in total, among which 5 via the Loop structure of the β-chain active site and 7 via the α-chain of ITGα5β1), comparing with that on ppAC (second row of Fig. 6F, 3 hydrogen bonds in total) and ppME (third row of Fig. 6F, 10 hydrogen bonds in total). The strong hydrogen bonding interactions contributed to the highest binding score and the best binding stability of ppAA group, thus showing the best activity.

#### Changes of intercellular signaling pathway

**Fig. 7:**
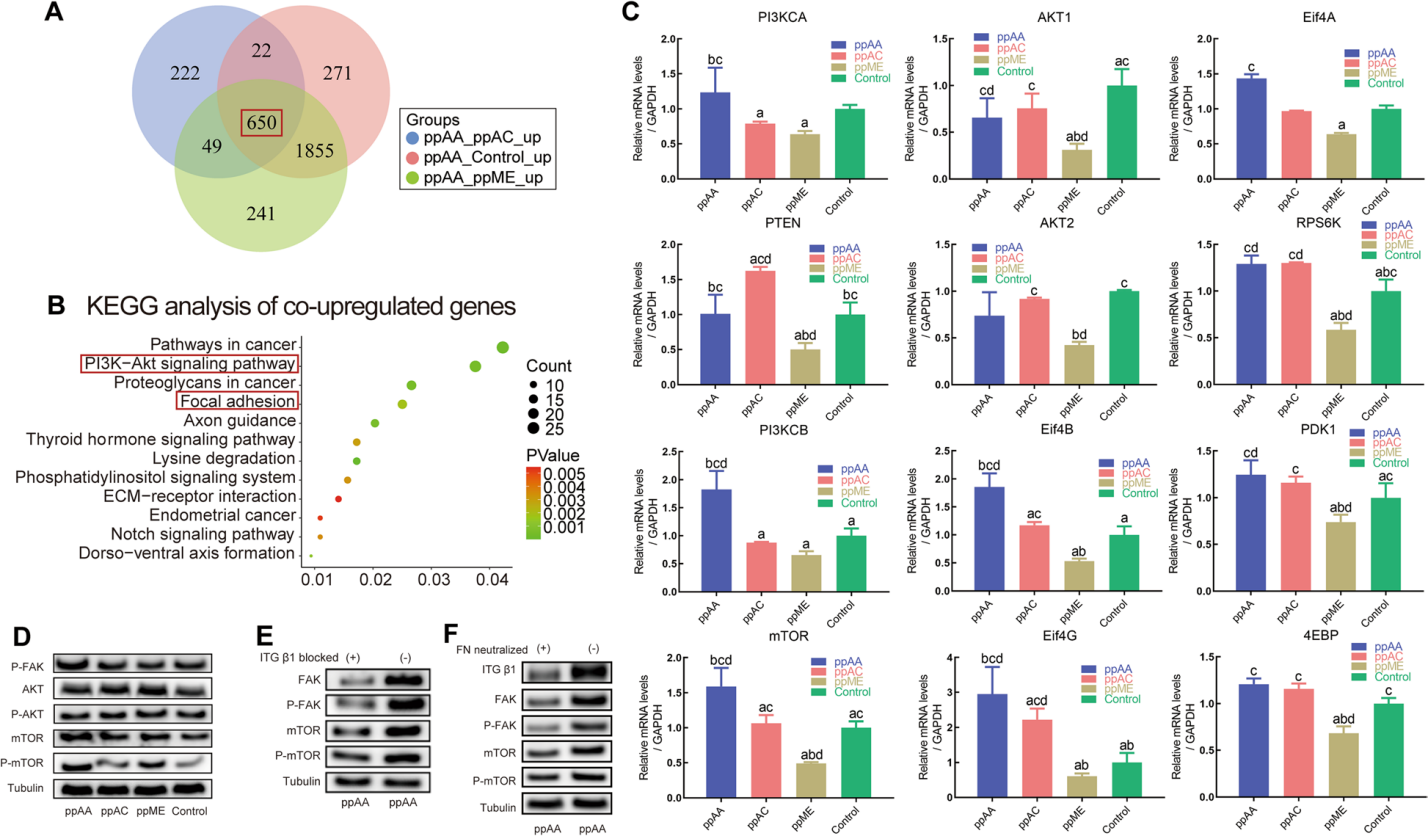
Changes in the intracellular signaling of HGEs cultured on the chemical surfaces. **A** Number of upregulated genes of HGEs on ppAA surface in comparison to the other surfaces; **B** KEGG analysis of the 650 types co-upregulated genes; **C** the expression of AKT-mTOR signaling pathway related genes by RT-qPCR; **D** representative western blot images of the FAK-AKT-mTOR signaling pathway related proteins; representative western blot images of FAK-mTOR signaling related proteins of HGEs cultured on ppAA surface with and without ITGβ1 blocking antibodies (**E**) and FN neutralize antibodies (**F**). Significant differences between two groups with a *p* < 0.05 are presented as follows: a) versus ppAA group, b) versus ppAC group, c) versus ppME group, and d) versus control group

To investigate the changes of intercellular signaling pathway, RNA Sequencing of HGEs cultured on the four surfaces was conducted, which showed distinct transcriptional profiles of each group (Figure S5). The Venn diagram of compared up-regulated proteins showed that there were 650 co-upregulated genes in the ppAA group compared with other groups (Fig. 7A). Further KEGG enrichment analysis suggested that these 650 types co-upregulated genes in ppAA group were enriched in epithelium barrier structure related terms such as focal adhesion and ECM-receptor interaction, PI3K-AKT signaling pathway, etc*.* (Fig. 7B).

Considering the FAK-mTOR signaling axis is important in regulating formation of epithelial barrier structure [38, 39], we detected the transcription and translation of the genes in this signaling pathway using RT-qPCR and western blotting (Fig. 7C and D). The results showed that the gene (*mTOR*) and proteins (including p-FAK, mTOR, p-mTOR), were mainly upregulated in the ppAA group. Meanwhile, the significantly up-regulated expression of downstream gene of mTOR signaling such as Eif4A, Eif4B, Eif4G, S6K, PDK1 and 4EBP in ppAA group further showed the activation mTOR pathway. Moreover, inhibition experiments using ITGβ1 blocking antibodies and FN neutralize antibodies showed that the activation of the FAK-mTOR signaling activation depended on FN and ITGβ1, thereby confirming the importance of FN-ITGβ1-FAK-mTOR in regulating ppAA mediated barrier structure formation (Fig. 7E and F).

#### Changes of cell adhesion behavior

Based on the superior absorption and functionalization capacity of FN on the ppAA surface, and the activation of FN-ITGβ1-FAK-mTOR adhesion related signaling, we set high expectations for the ppAA chemical surface to increase epithelium barrier structure formation and improve epithelial cell adhesion.

**Fig. 8:**
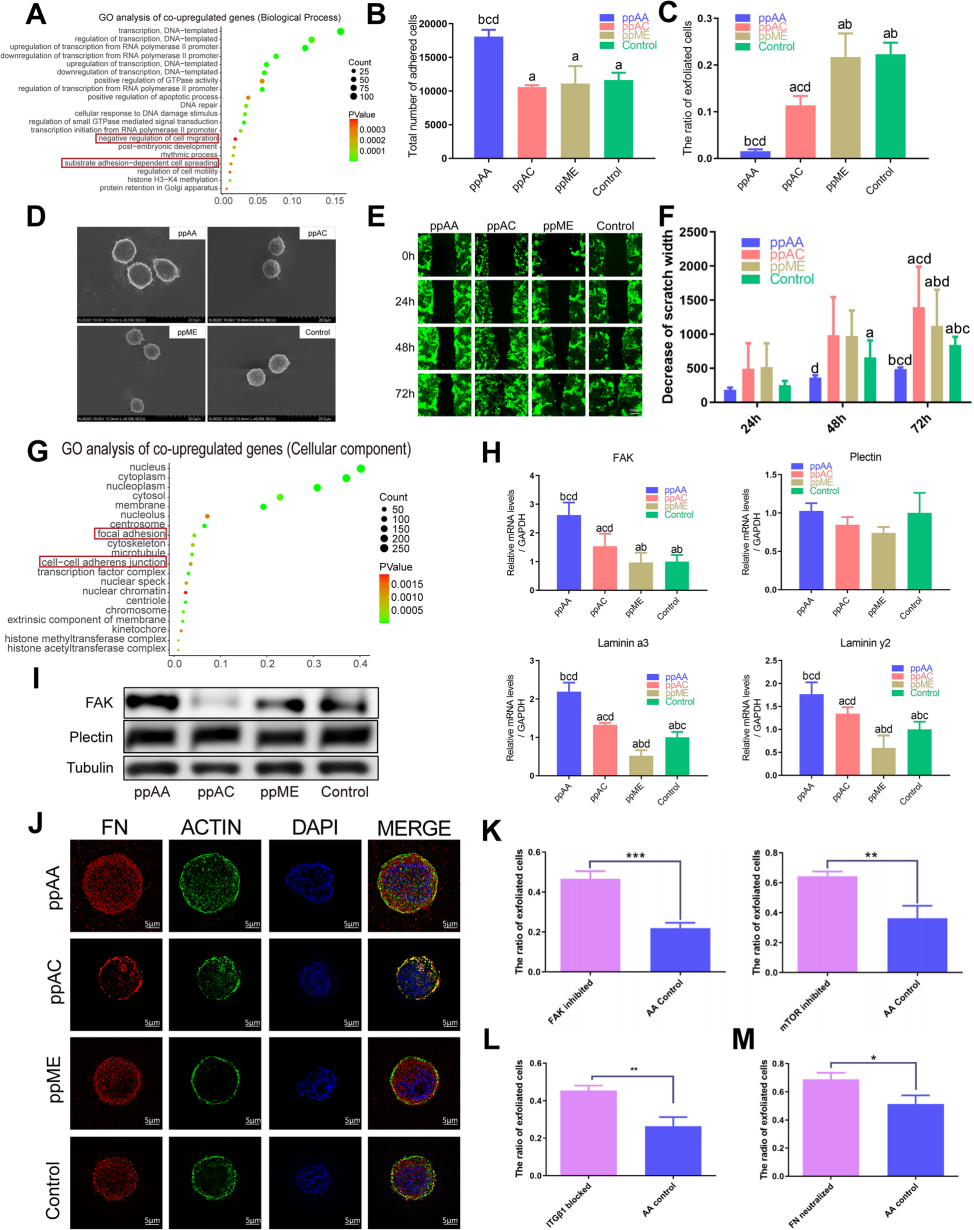
Changes in the adhesion behaviors of HGEs cultured on the chemical surfaces. **A** GO enrichment analysis of the 650 types co-upregulated genes on the biological process level; **B** Number of remined adhered HGEs cultured on four groups of surfaces; **C** Ratio of exfoliated HGEs cultured on four groups of surfaces; **D** Representative SEM images of spreading morphology of HGEs cultured on four groups of surfaces; **E** The representative images and (**F**)semi-quantitative statistical analysis of HGEs migration on four groups of surfaces during a wound healing assay; **G** GO enrichment analysis of the 650 types co-upregulated genes on the cellular component level; **H**) RT-qPCR results and (**I**) representative western blot images results of the expression of focal adhesion and hemidesmosome related genes and proteins; **J** SIM immunofluorescence showed the FN adsorption together with HGEs cytoskeleton on four surfaces; ratio of exfoliated HGEs cultured on ppAA surface with and without FAK and mTOR inhibitors (**K**), ITGβ1 blocking antibodies (**L**) and FN neutralize antibodies (**M**). Significant differences between two groups with a *p* < 0.05 are presented as follows: a) versus ppAA group, b) versus ppAC group, c) versus ppME group, and d) versus control group. *: *p* < 0.05, **: *p* < 0.01, ***: *p* < 0.001

GO enrichment analysis suggested that these 650 types of co-upregulated genes in ppAA group were related with the regulation of cell spreading and cell migration in biological process level (Fig. 8A), and focal adhesion in cellular component level (Fig. 8G). We further conducted a cell shedding test and found that the ppAA group had significantly less exfoliated cells with more cells remained adhered on the ppAA surface after the shedding process, as compared with the other three groups (Fig. 8B&C), which indicated improved cell adhesion. Furthermore, we used scanning electron microscopy (SEM, Fig. 8D) and cell migration experiments (Fig. 8E&F) which confirmed the larger spread morphology and improved anchoring of human gingival epithelial cells on the ppAA surface over other groups, thus verifying the improved cell-substratum adhesion ability of HGEs on the ppAA surface. As focal adhesion and hemidesmosomes are important epithelium barrier structures, [6] we further evaluated their related molecules. The expression of focal adhesion kinase (FAK) was significantly higher on the ppAA surface, as detected by RT-qPCR (Fig. 8H) and WB (F ig. 8I), while the gene and protein expression of plectin showed no significant differences among four groups, indicating that the ppAA surface promote HGEs adhesion via upregulating the formation of focal adhesion. The FN adsorption together with HGEs cytoskeleton were analysis by the Structured Illumination Microscopy (SIM). The immunofluorescence results showed that HGEs on the ppAA surface exhibited the best spreading morphology accompanied with the most amount of FN adsorbed surrounding the cell (Fig. 8J).

Moreover, the results of cell adhesion assay after the inhibition of FAK and mTOR [40] showed that the FAK-mTOR pathway was correlated to the adhesion of HGEs to ppAA (Fig. 8K). The blockade of membrane receptor ITG β1 [40, 41] inhibited the adhesion of HGEs to the ppAA surface and downregulated the protein expression of the adhesion-based FAK-mTOR pathway (Fig. 8L and 7E). Utilizing FN neutralizing antibodies that specifically bind to the RGD sequence [42] significantly inhibited the adhesion of HGEs to ppAA and downregulated the protein expression of ITG β1 and adhesion-based FAK-mTOR pathway (Fig. 8M and 7F). The above results suggest that ppAA surface can activate ITG β1-FAK-mTOR signaling cascade to regulate the adhesion of HGEs by adsorbing FN and mediating the exposal of its RGD site, thus indicating the potential of ppAA to enhance the formation of epithelial barrier structure.

As for the establishment of epithelial barrier, the cell-to-cell interaction is also important because it guarantees the continuity of the epithelial barrier. Interestingly, GO CC enrichment of the co-upregulated genes in ppAA compared to other group showed that cell–cell adherens junction related genes were enriched (Fig. 8G). Further GO BP analysis of the cell–cell adherens junction related genes indicated close relationship to cell–cell junction organization (Figure S5C), among which CDH1, BMPR2, SMAD7, DLG5 genes are the main contributors [43–46], suggested by PPI analysis (Figure S5D). Then, their mRNA expressions were verified by RT-qPCR assay (Figure S5E). These results indicated that ppAA surface could not only promote the HGEs to firmly adhere to substrate but also induce the cell–cell junction to enhance a strong epithelial barrier. Further study of transepithelial medical device should put in more effort to improve cell–cell junction to create a firm epithelial barrier structure.

## Discussion

In the study, plasma polymerization was utilized to accurately and controllably generate chemical coatings possessing strong and stable interaction capacity with adsorbed proteins. As the -NH_2_, -COOH, -CH_3_ groups are commonly found in proteins and have been shown to possess the potential in triggering distinct fibronectin adsorption behaviors, we therefore chose to use these three precursors (allylamine, acrylic acid, and 2-methyl-oxazoline) to generate plasma polymerized model surfaces for investigation of their effect on fibronectin adsorption. The physicochemical characterization showed that the plasma polymerized technique successfully generated desired chemical surfaces by simply choosing the proper reaction precursors and tuning the work parameters. Such characteristic could favor its transfer to different medical surfaces and facilitated the study of the influence of surface chemistry on adsorbed proteins and subsequent cellular responses in the present study.

The amount and conformational exposure results confirmed that ppAA surface has the superiority over ppAC and ppME in capturing and functionalize FN from the profile of adsorbed serum proteins, and might favor the formation of epithelial barrier structure on ppAA surface. It should be noted that the capacity of plasma polymerized coating to adsorb protein is a combine result of all its surface physicochemical properties. Despite of surface chemistry, other surface properties, *e.g.,* surface roughness and the thickness of the surface polymer film, could also affect protein adsorption. Studies found that thicker polymer film could be more resistant to protein adsorption, which might be explained by the longer length of the polymer chains [47, 48]. Interestingly, in our study, although ppAA exhibited the greatest thickness among the three surfaces (Fig. 2 E), it was still the most capable one to capturing FN (Fig. 4C&F). We assumed that it was because the chemical property was more dominant than thickness in the present occasion of plasma polymerized surface-fibronectin binding. Studies have explored the influence of surface roughness on protein adsorption and found that surface roughness ranging from several to tens of nanometers of roughness could significantly affect the adsorption of FN [49–53]. Therefore, we managed to fabricate the three plasma polymerized chemical surfaces with sub-nanometer roughness (Fig. 2F) to minimize the confounding impact of surface roughness on the result of the current study.

The molecular dynamics simulation analysis demonstrated that ppAA chemical surface exhibited superior FN adsorption capacity owing to its enhanced affinity to the FN molecule, which was determined from the time of contact, and the type and stability of intermolecular forces between the protein and the ppAA surface (Fig. 5H). Despite both ppAA and ppME surface could induce a close proximity of RGD and PHSRN sites, the ppAA surface could form intermolecular forces to induce a more sufficient and stable functionalization of FN that promoted cell adhesion. The ppAA surface could also form intermolecular forces to induce a good functionalization that favor promoting cell adhesion, including full exposal and close proximity of RGD and PHSRN sites. Such molecular scale insight of the FN’s adsorption onto pp surfaces further convinced us of the potential of ppAA to enhance the formation of epithelial barrier structure.

**Fig. 9:**
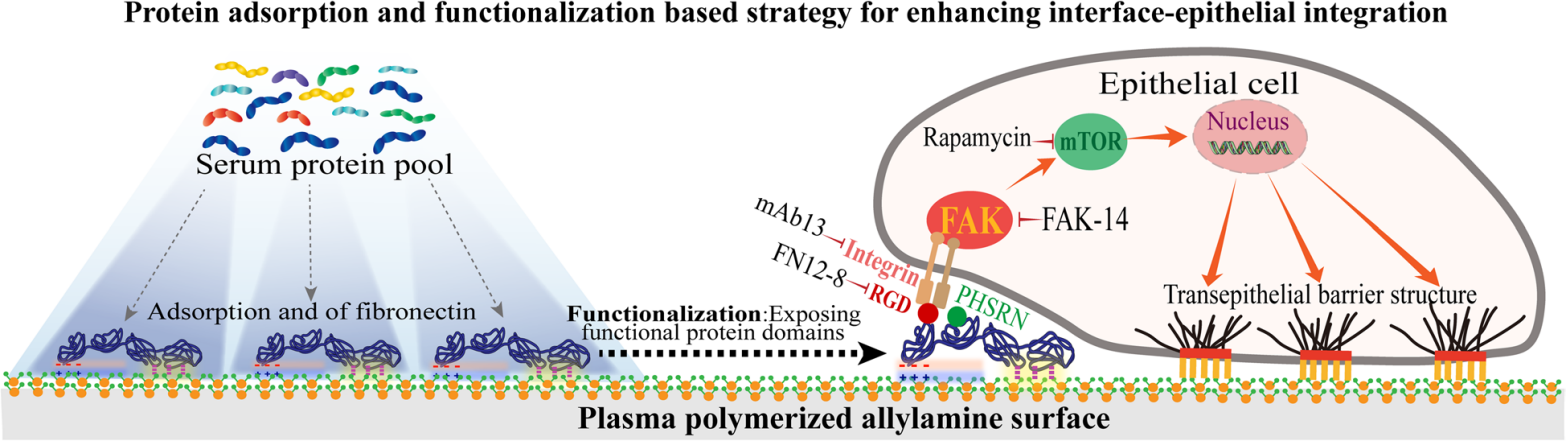
Schematic figure of the protein adsorption and functionalization-based strategy for enhancing interface-epithelial integration. Plasma polymerized allylamine surface is capable of adsorbing high amount of fibronectin from the serum protein pool and functionalizing it by exposing its functional protein domains. The functionalized fibronectin can strongly bind to cell membrane integrin and activate epithelial barrier structure related signaling pathway to enhance the formation of epithelial barrier structure, which provides a good strategy for improving interface-epithelial integration

In this study, our results established that the ppAA surface could modulate in situ FN absorption and functionalization to activate the ITG β1-FAK-mTOR signaling and induce the formation of epithelial barrier structures, thus offered an effective perspective to enhance interface-epithelial integration (Fig. 9). This confirms the feasibility of the in situ protein absorption and functionalization based strategy for improving the “epithelial barrier structures” formation, thus solving the discontinuous epithelial barrier led by transepithelial devices. With the feasibility of plasma polymerization technique to generate specific chemical coatings in a substrate-independent manner, hopefully it can be applied onto different transepithelial medical devices in various medical situations that penetrate skin or mucosa, including but not limited to peritoneal dialysis catheters penetrating abdomen skin, rigid external fixation penetrating the of skin of fractured limbs, needle-type glucose sensors or drug delivery micro-needle patch penetrating arm skin, and dental implants penetrating oral gingiva [6, 54–56].

In addition to surface chemistry, the precise effect of different specific physicochemical surface parameters (surface charge, topography, hydrophilicity, etc*.*) in inducing a corresponding in situ proteins modulation and its further effects on interface-epithelial barrier structure formation deserves more explorations for to boost the efficiency and effectiveness of transepithelial devices development. The in situ protein mediated regulation of interface-epithelial barrier structures formation are complicated and far from well understood. Moreover, epithelial cell–cell junction formation is also important. To further develop this strategy, the effect of in situ modulation of diverse barrier structures-related proteins (laminins, collagens, thrombospondins, etc*.*) and their coordinated combinations on interface-epithelial integration should be thoroughly investigated.

It should be noted that in addition to the ability to guide the adsorption of serum proteins and formation of epithelial barrier structure, the antibacterial and antifouling properties of transepithelial bio-interface should not be forgotten. Although the limited antibacterial activity of chemical groups of the monomers might hinder the antibacterial efficiency of plasma polymerized surfaces per se, it can serve as carrier matrices for antibacterial agents as a reservoir for the out-diffusion of antibacterial ions [57]. The anti-fouling property of plasma polymerized coatings is gaining attention but still need further study [58]. Also, the retentivity and durability of plasma polymerized surface coating should be considered because of the challenging occasions that transepithelial medical devices are faced with in body environment. Future ideal transepithelial medical device should own an interface not only enhancing the formation of epithelial barrier structure but also exhibiting antibacterial and antifouling ability while maintaining good retentivity and durability. Moreover, as different cells, *e.g.,* Langerhans cell, Merkel cell, stem cells and leucocytes, present around the epithelial barrier on plasma polymerization biointerface coatings [59, 60], their effects on the coatings should also be concerned. Optimized plasma polymerization coatings are biocompatible and remain stable when facing surrounding cells, while adsorption of cytokines and extracellular vesicles produced by surrounding cells can endow the coatings with immunomodulatory and tissue regenerative compacity [61–64]. In terms of this, more studies are need more further studies to better utilize this technique for enhancing the establishment of epithelial barrier.

## Conclusions

The generated plasma polymerized surfaces could successfully direct distinguished protein adsorption profiles, in which ppAA surface favors adsorbing adhesion related proteins and can modulate FN adsorption behaviors via electrostatic interactions and hydrogen bonds, thus subsequently activating the ITG β1-FAK-mTOR signaling and promoting epithelial cell adhesion. The study offered an effective perspective to enhance bio-interface-epithelial integration to meet the significant clinical demands by in situ protein absorption and functionalization.

## Experimental section

### Preparation of chemical surfaces

All monomers allylamine (AA), acrylic acid (AC) and methyl-oxazoline (ME) used for plasma polymerization were purchased from Merck—Sigma Aldrich, Australia. Tissue culture plates and silicon wafers were used as substrates. A custom-made plasma reactor was used to perform plasma polymerization on these substrates. First these substrates were cleaned by sonicating them in acetone and ethanol and then air cleaned by varying the plasma parameters (Power: 50 W; Pressure: 0.1 mbar and Time: 5 min). Finally, the substrates were coated with ppAA, ppAC and ppME by using different plasma parameters as described in Table S1. The schematic representing the plasma polymerization process for fabricating different coatings has been described in Figure S1A.

### Physicochemical characterization of the one-parameter chemical surface

X-Ray Photoelectron Spectroscopy (XPS) was performed to evaluate the elemental composition. Survey was recorded using a Spec SAGE XPS over 0—1000 eV range, pass energy of 100 eV and resolution of 0.5 eV. C1s peak of carbon at 285 eV was utilized as the reference to calibrate all binding energies. Elemental composition from survey spectra and fitting of C1s curve was performed using Casa XPS software. The thickness of the plasma polymer coatings was evaluated by a variable angle spectroscopic ellipsometer (J. A. Woollam Co. Inc.). Firstly, WVASE32 software was used to calibrate the system. Then, the measurement was performed over a range of wavelengths (250 to 1100 nm) and the data was collected at different angles (65º, 70º and 75º). Finally, the data collected was analyzed by fitting in Cauchy model. The final thickness was reported by measuring a minimum of 9 samples. The wettability of different chemistries fabricated on these surfaces were evaluated by a contact angle goniometer. 10 µl water droplets (3 drops) were carefully placed on the plasma coatings and their images were adsorbed immediately. Finally, the images were analyzed using “Drop Snake plug-in” in an Image J software. Zeta potential of different plasma modified surfaces was measured using Zetasizer Nano ZS (Malvern, UK) and Smoluchowski equation (MÜLLER, 1191) was used to transform this into zeta potential for recognition. 10^–3^ M KCl was utilized to measure zeta potential of all the coated samples. The surface topography and roughness of different coatings were measured using an Atomic Force Microscopy (AFM). Gold coated silicon nitride tips with resonance frequencies between 65 to 100 kHz with a spring constant between 0.35- 6.06 N/m were used. A scan rate of 0.5 Hz and amplitude of 10 nm was used to scan 2 µm × 2 µm images. Surface roughness was then obtained from these images using WSXM software.

### Preparation of absorbed protein on four chemical surfaces for LC–MS/MS

The 24-well plates with different chemical surfaces (ppAA, ppAC, ppME) and normal tissue culture plates (TCP) were incubated in complete culture medium (Dulbecco’s modified Eagle medium (DMEM) supplemented with 10% (V/V) fetal bovine serum) at the cell incubator for 1 h and then rinsed twice with 500μL PBS. The species (bovine) and concentration (10%) of serum was determined in regard of in vitro cellular incubation condition, according to similar studies [30, 65]. 50μL 2% SDS/PBS was added to each well for 5 min. The protein adsorbed by the four chemical surfaces was collected by scratching the surfaces with 10μL pipette tips. The protein concentrations of the samples were determined using BCA assay and equal amount of protein from each sample was taken. After adjusting the solution concentration by adding ammonium bicarbonate and dithiothreitol, the samples were incubated for 30 min at 60 °C. Subsequently, iodoacetamide was added and the samples were incubated in the dark for 30 min. The solution digestion was performed using trypsin at 37 ℃ for 12–16 h. Following the digestion, the samples were diluted to 50 mM NH_4_HCO_3_ and then acidified with TFA. After centrifuged to remove SDC, the samples were added to C18 column and salts were cleared. The samples were reconstituted in 0.1%FA after freeze-dry processing for LC–MS/MS.

### LC–MS/MS experiment

For mass spectrometry analysis, the samples were separated via a 90 min gradient elution using a Thermo Scientific EASY-nLC 1000 HPLC system. The samples were derivatized with a precolumn at a rate of 220 nl/min and then flushed onto an analytical RSLC column and eluted with 0.1% formic acid in water and 0.1% formic acid in acetonitrile. MS acquisition was operated with Xcalibur 2.2 SP1 and the scan parameters were set as follow: the scan range of precursor ions 300-2000 m/z, scan resolution 70,000. The 20 most intensive peptide signals were selected and the raw data were obtained by higher energy collisional dissociation fragmentation using a collision energy of 27%.

### Database search of LC–MS/MS data

Database search and relative quantitative analysis were processed within the MaxQuant environment (version 1.6.0.1) and all annotations were extracted from the UniProt database. Peptide identification was performed with an initial precursor mass deviation up to 7 ppm and a fragment mass tolerance of 0.05 Da. Search results were filtered by stringent criteria (PeptideFDR ≤ 1%, ProteinFDR ≤ 1%). The original MS/MS file data were submitted to Maxquan Software (Version 1.6.0.1) for analysis. Three samples were analyzed in each group. The valid data produced by MaxQuant was filtered and further analyzed using the corresponding iBAQ and LFQ intensity [66, 67].

### Bioinformatics analysis

Hierarchical clustering analysis by R-package “pheatmap” was utilized for all detected protein and differential screening protein. The Gene Ontology (GO) enrichment analysis were performed on three levels of biological process (BP), cellular component (CC), and molecular function (MF) by utilizing the Database for Annotation, Visualization and Integrated Discovery (DAVID; https://david.ncifcrf. gov/summary.jsp). *P*-value < 0.05 was regarded as significant. The STRING online database (http://www.string-db.org/) was utilized to calculate protein–protein interaction (PPI) scores. The visual network was constructed using Cytoscape and the modules were identified with the Mcode plugin. *P* value < 0.05 was regarded as significant.

### Molecular dynamics simulation of fibronectin absorbed onto chemical surfaces

The material layer was constructed with polyallylamine, polyacrylicacid and polymethyl-oxazoline, respectively. Then, polyallylamine layer (ppAA), polyacrylicacid layer (ppAC), and polymethyl-oxazoline layer (ppME) were constructed by PACKMOL, with a size of 20 × 15 nm, in which the amino group on the ppAA branched chain was protonated and positively charged, while the carboxyl group on the ppAC branched chain was deprotonated and negatively charged. The protein structure of FNIII_7-10_ is derived from PDB database, PDB ID is 1FNF. The adsorption of FnIII_7-10_ protein on surfaces was studied by Gromacs5.0.4 program, and its kinetic behavior in solvent state was evaluated. In the process of kinetic simulation, the OPLS force field is used for layer and the SPC model is used for water molecules.

The system energy optimization was performed using the conjugate gradient and steepest descent method. The NVT and NPT equilibrium were carried out. The simulation process persisted for 60 ns. The (leapfrog algorithm), integral step of the leapfrog algorithm is set to 2 fs, the PME algorithm is used to deal with the long-range electrostatic interaction, and the short-range Coulomb truncation radius is set to 1.2 nm. The truncation radius of van der Waals interaction is 1.2 nm. In the process of simulation, the material layer is fixed in the initial position, the system adopts periodic boundary conditions in all directions, and the bond length is constrained by LINCS algorithm. The simulation results are analyzed by Gromacs5.0.4 and visualized by PyMol.

### Molecular docking simulation

FN-biointerface/ITGα5β1-membrane docking simulation study were performed using HDock. Residues 1491–1498 of the FN protein were set as amino acids participating in the interaction (Receptor/Ligand Binding Site Residues) and the prediction of the transmembrane structure of ITGα5β1 protein were performed (Figure S6). All possible spatial conformations and interaction patterns were searched, and the lowest energy conformation was selected for visual analysis using PyMolv1.60 and VMD software.

### Cell culture

Human gingival epithelial cells (HGEs) were purchased from Shanghai Bioleaf Biotech Co. Ltd for experiment. Cells were cultured in complete culture medium (Dulbecco’s modified Eagle medium (DMEM) supplemented with 10% (V/V) fetal bovine serum) (Thermo Scientific) at 37 °C in an atmosphere of 5% CO_2_, which was replaced every 2–3 days. The cells in the culture flask were passaged using trypsin when they reached about 80% confluence with the passage ratio of 1: 4.

### ELISA assay of absorbed FN on different surfaces

The FN ELISA kit was purchased from RayBio Co. Ltd. 24-well plate with different chemical surface (3 wells for each chemical surface) coating was incubated in 0.5 ml of FBS for 1 h. Then they were washed gently with 1 ml PBS, after which ELISA assay was carried out refer to the attached instructions.

### Human gingival epithelial cells adhesion force assay on different chemical surfaces

Well-grown HGEs were digested using trypsin, and the obtained cells were seeded on material surfaces of four groups (ppAA, ppAC, ppME and Control) at a density of 2.5 × 10^4^ cells/well in cell incubator for 1 h. After gently rinsing twice with PBS, the plates were continuously shake for 15 min at 37℃ (200 rpm), and the shaken culture mediums were collected. Each well was added with 500μL trypsin and continuously shaken for 15 min at 37℃ (200 rpm), then the shaken digestion was added with an equal volume of complete culture medium to terminate the reaction and collected. The number of cells contained in two cell suspensions were counted using a fully automated cell counter. The adhesion force was indicated by the total number of adhered cells and non-adhesion rate. The cells were cultured on ppAA chemical surface with or without FN_12-8_ (FN inhibitor, Takara), mAb13(ITGβ1 inhibitor, Cell Signaling Technology), FAK inhibitor 14 (FAK inhibitor, Sigma-Aldrich) and Rapamycin (mTOR inhibitor, Cell Signaling Technology) and underwent the above-mentioned shedding assay.

### Cell viability and proliferation test

Well-grown HGEs were digested using trypsin, and the obtained cells were seeded on material surfaces of four groups (ppAA, ppAC, ppME and Control) at a density of 2 × 10^3^ cells/well in cell incubator. After 6, and 24 h, cell viability and proliferation assays were performed using the Cell Counting Kit-8 (CCK-8; Dojindo).

### Cell apoptotic rate analysis

For cell apoptotic rate analysis, we prepared HGEs of each group in 100 μl suspension. Then HGEs were stained with 1 μl Annexin V and 7AAD antibody at room temperature for 30 min. The flow cytometry analysis was performed using flow cytometry (ACEA NovoCyteTM).

### RNA sequencing

Well-grown HGEs were digested using trypsin, and the obtained cells were seeded on material surfaces of four groups (ppAA, ppAC, ppME and Control) at a density of 10^6^ cells/well in cell incubator for 1 h. The samples were gently rinsed twice with PBS and the total RNA from HGEs was extracted. mRNAs with polyA tails were enriched by magnetic beads with OligodT. The obtained RNA was fragmented, reverse transcript and amplified. The RNA sequencing (RNA-Seq) was performed by Huada Gene Company (Shenzhen, China) using BGIseq500 platform.

### Human gingival epithelial cells migration on different chemical surfaces

*GFP transfection in HGEs*: Well-grown HGEs were digested using trypsin, and the obtained cells were seeded on tissue culture plate at a density of 2.5 × 10^4^ cells/well and incubated for 24 h before GFP transduction. The number of HGEs at the time of lentivirus transfection was about 2 × 10^5^ per well. The culture medium was replaced by 300 μl fresh DMEM supplemented with 6 μg/ml polybrene. The virus-containing supernatants was introduced into the cells and incubated for 4 h at 37℃. Then, 300 μl fresh DMEM were added to diluted polybrene before incubation for 24 h. The medium was replaced with fresh complete culture medium for further incubation. Cells with successful GFP transfection were collected by flow cytometry and subjected to cell culture and expansion.

### Cell migration experiment

Three groups of plasma polymer-coated modified coverslip (ppAA, ppAC, ppME) and common coverslip (Control) were placed in the confocal laser scanning dish. The healing insert dedicated to cell migration assay was tightly pressed in the center of the coverslip, and 5 × 10^3^ well-grown HGEs (transfected with GFP) were seeded in the septa on both sides of the insert and cultured in cell incubator. When the cell growth in the septa reached a confluence of about 80%, the healing insert was vertically removed to form a standard-width cell-free area on the material surface of each group. After rinsing twice with PBS, the culture medium was replaced. The cells were imaged under an invert fluorescent microscope at 0, 24, 48 and 72 h.

### Cells spreading observation

Well-grown HGEs were digested using trypsin, and the obtained cells were seeded on three groups of plasma polymer-coated modified coverslip (ppAA, ppAC, ppME) and common coverslip (Control) at a density of 10^5^ per well. After incubated for 1d, the HGEs were rinsed two times with PBS and fixed by 3% paraformaldehyde at 4℃ overnight. Subsequently, the specimens were dehydrated using an ascending series of alcohol. After critical point drying and gold coating, cell morphologies were observed and recorded using SEM.

### The evaluation of adhesion related genes and proteins

Well-grown HGEs were digested using trypsin, and the obtained cells were seeded on material surfaces of four groups (ppAA, ppAC, ppME and Control) at a density of 10^6^ cells/well in cell incubator for 1 h. The samples were gently rinsed twice with PBS. The total RNA from HGEs was extracted to detect the expression levels of adhesion-related genes *FAK, Plectin, ITG β1* (Table S2). Western blot was used to detect the expression of adhesion-related proteins, including FAK (1:1000, Cell Signaling Technology), Plectin (1:1000, Abcam) and Integrin β1 (1:200, Abcam). Immunofluorescence staining was conducted to detect the protein expression of FAK (1:200, Abcam) and Integrin β1 (1:200, Abcam).

### The evaluation of FAK-mTOR signaling related genes and proteins

HGEs were digested using trypsin, and the obtained cells were seeded on material surfaces of four groups (ppAA, ppAC, ppME and Control) at a density of 10^6^ cells/well in cell incubator for 1 h. The samples were gently rinsed twice with PBS. The total RNA from HGEs was extracted to detect the expression levels of *PI3KCA, PI3KCB, mTOR, AKT1, AKT2, PTEN, Eif4A, Eif41B, Eif4G, RPS6K, PDK1, 4EBP* (Table S2). Western blot was used to detect the expression of adhesion-related proteins, including P-FAK (Tyr397, 1:1000, Cell Signaling Technology), AKT (1:1000, Cell Signaling Technology) and P-AKT (Thr308, 1:1000, Cell Signaling Technology), mTOR (1:1000, Cell Signaling Technology) and P-mTOR (Ser2448, 1:1000, Cell Signaling Technology). The cells were further cultured on ppAA chemical surface with or without FN_12-8_ (FN inhibitor, Takara) and mAb13(ITGβ1 inhibitor, Cell Signaling Technology), and underwent the above-mentioned western blot assay to detect the protein expression of Integrin β1, FAK, P-FAK, mTOR and P-mTOR.

### Statistical analysis

Graph pad prism 8 software were used to perform graphs and statistics and presented as mean (SD). The one-way ANOVA followed by Tukey’s multi comparison test was utilized to demonstrate statistical significance. *P* < 0.05 was considered statistically significant.

## Supplementary Information

**Additional file 1: Figure S1.** A) The manufacturing process of plasma polymerized surface. Plasma polymerization was performed on ultrasonic-cleaned, air-dried tissue culture plates or coverslips using a custom-built plasma reactor equipped with a 13.56 MHz plasma generator. Substrates were coated by allylamine (AA), acrylic acid (AC) or methyl-oxazoline (ME) monomers by using different plasma parameters (Table S1). B) Cell viability of HGEs cultured in four groups detected by CCK8 assay. X-axis indicates the time after seeding cells. C) Flow cytometry of HGEs in the four groups using the apoptotic marker Annexin V (left) and quantification (right).

**Additional file 2: Figure S2.** Protein adsorption profiles of the four surfaces. A) Heatmap analysis of the adsorbed proteins on each surface showing general intragroup similarity; B) GO enrichment analysis of the total adsorbed proteins on each surface showing extensive regulatory potentials; cluster heatmap of differential adsorbed proteins of ppAA versus control (C), ppAC versus control (D) and ppME versus control (E). The control group, as mentioned in the experimental section, referred to adsorbed proteins on tissue culture plate without plasma polymer modification.

**Additional file 3: Figure S3.** Establishment of the molecular dynamics simulation system. A) 3D diagram of the key fragment of the FN molecule, where the red and blue indicators show the positively and negatively charged areas, respectively. FN III_7-10_ molecule floating above the chemical surface in the environment absent of solvent (B) and with solvent (C); Root Mean Square Deviation (RMSD) (D) and Solvent Accessible Surface Area (SASA) analysis (E) of FN III_7-10_ on the three surfaces during molecular dynamic simulation process. F) The distance between the amino acid residues of FN that formed hydrogen bonds with ppAA surface, and their alterations over time.

**Additional file 4: Figure S4.** A) Fluctuation curve of the secondary structure of the FN molecule on the three chemical surfaces indicating the secondary structural alterations of the fibronectin during the molecular dynamics simulation; B-D) structural superposition analysis of FN in ppAA (B), ppAC (C), ppME (D) groups at different time points; E) the three-dimensional structure diagram of ITGα5β1 receptor protein in cell membrane; F) the optimal binding conformation of the FN-pp/ITGα5β1-membrane complex.

**Additional file 5: Figure S5.** A) Principal component analysis of gene expression of HGEs on four surfaces; B) Volcano plot showed the differential expressed genes between the groups; C) Gene Ontology - Biological Process analysis of the co-upregulated cell-cell adherens junction related genes in ppAA group revealed a close relationship with the promotion of cell-cell junction organization; D) Protein-protein interaction anylysis of cell-cell adherens junction genes co-upregulated in ppAA compared with other groups. Dot size and color depth indicate connectivity in the network; E) The relative mRNA expression of CDH1, BMPR2, SMAD7, DLG5 genes in each group, detected via RT-qPCR assay.

**Additional file 6: Figure S6.** Prediction of the transmembrane structure of α5 chain (A) and β1 chain (B) of ITGα5β1 protein.

**Additional file 7: Table S1.** Deposition conditions for plasma polymerization of allylamine (AA), acrylic acid (AC) and methyl-oxazoline (ME).

**Additional file 8: Table S2.** Primer used in the RT-qPCR assays.

**Additional file 9: Video 1.** The dynamic adsorption process of FN onto ppAA surface.

**Additional file 10: Video 2.** The dynamic adsorption process of FN onto ppAC surface.

**Additional file 11: Video 3.** The dynamic adsorption process of FN onto ppME surface.

## Data Availability

The datasets used and/or analysed during the current study are available from the corresponding author on reasonable request.

## Abbreviations

AA: Allylamine
AC: Acrylic acid
ME: 2-Methyl-oxazoline
pp: Plasma polymerization
TCP: Tissue culture plate
LC–MS/MS: Liquid Chromatography-Tandem Mass Spectrometry
PPI: Protein–protein interaction
MS: Molecular dynamics simulation
HGEs: Human gingival epithelial cells
ITG: Integrin
WB: Western blotting
RT-qPCR: Real-time quantitative polymerase chain reaction
FAK: Focal adhesion kinases
FN: Fibronectin
SEM: Scanning electron microscopy
SIM: Structured Illumination Microscopy
XPS: X-Ray Photoelectron Spectroscopy
AFM: Atomic Force Microscopy
DMEM: Dulbecco’s modified Eagle medium
GO: Gene Ontology
BP: Biological process
CC: Cellular component
MF: Molecular function
DAVID: Database for Annotation, Visualization and Integrated Discovery
RNA-Seq: RNA sequencing
