# Supplementary figures and images for: Plasma polymerized bio-interface directs fibronectin adsorption and functionalization to enhance “epithelial barrier structure” formation via FN-ITG β1-FAK-mTOR signaling cascade
Source: Biomater Res. 2022 Dec 26;26:88. doi: 10.1186/s40824-022-00323-0 (PMC9791785; doi:10.1186/s40824-022-00323-0)

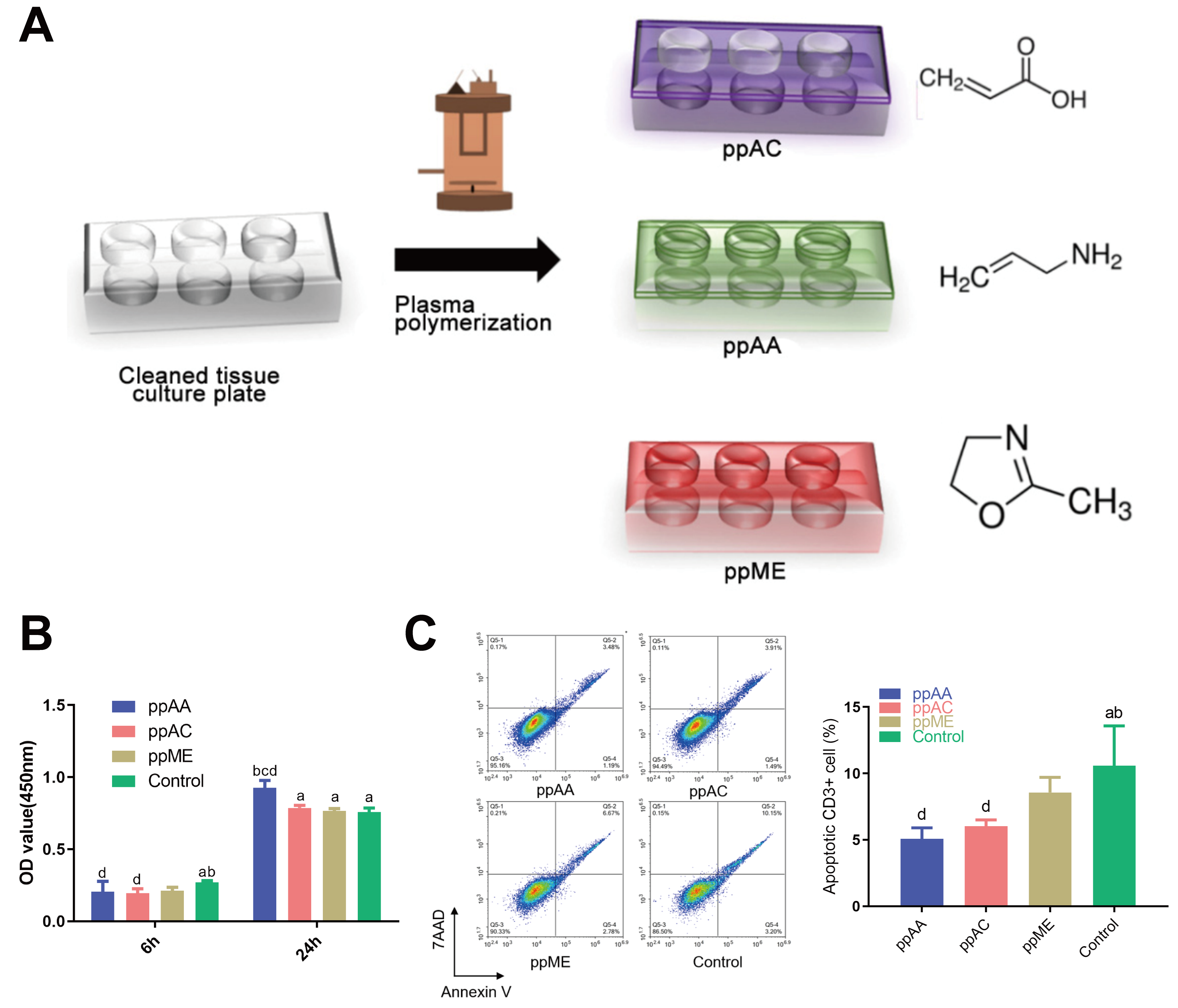

Supplement: Supplementary file 1 — Additional file 1: Figure S1. A) The manufacturing process of plasma polymerized surface. Plasma polymerization was performed on ultrasonic-cleaned, air-dried tissue culture plates or coverslips using a custom-built plasma reactor equipped with a 13.56 MHz plasma generator. Substrates were coated by allylamine (AA), acrylic acid (AC) or methyl-oxazoline (ME) monomers by using different plasma parameters (Table S1). B) Cell viability of HGEs cultured in four groups detected by CCK8 assay. X-axis indicates the time after seeding cells. C) Flow cytometry of HGEs in the four groups using the apoptotic marker Annexin V (left) and quantification (right). [file 40824_2022_323_MOESM1_ESM.tif]

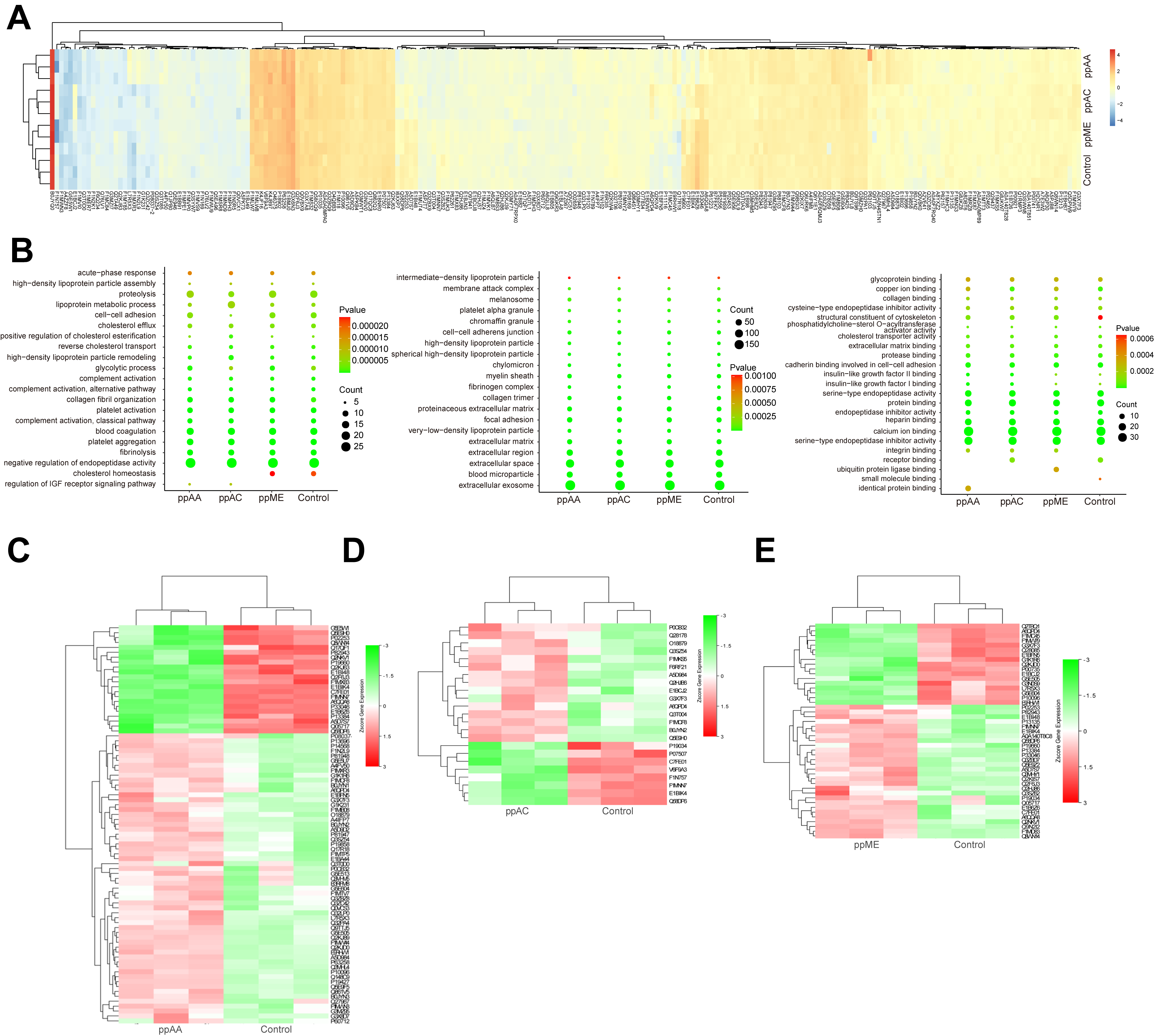

Supplement: Supplementary file 2 — Additional file 2: Figure S2. Protein adsorption profiles of the four surfaces. A) Heatmap analysis of the adsorbed proteins on each surface showing general intragroup similarity; B) GO enrichment analysis of the total adsorbed proteins on each surface showing extensive regulatory potentials; cluster heatmap of differential adsorbed proteins of ppAA versus control (C), ppAC versus control (D) and ppME versus control (E). The control group, as mentioned in the experimental section, referred to adsorbed proteins on tissue culture plate without plasma polymer modification. [file 40824_2022_323_MOESM2_ESM.tif]

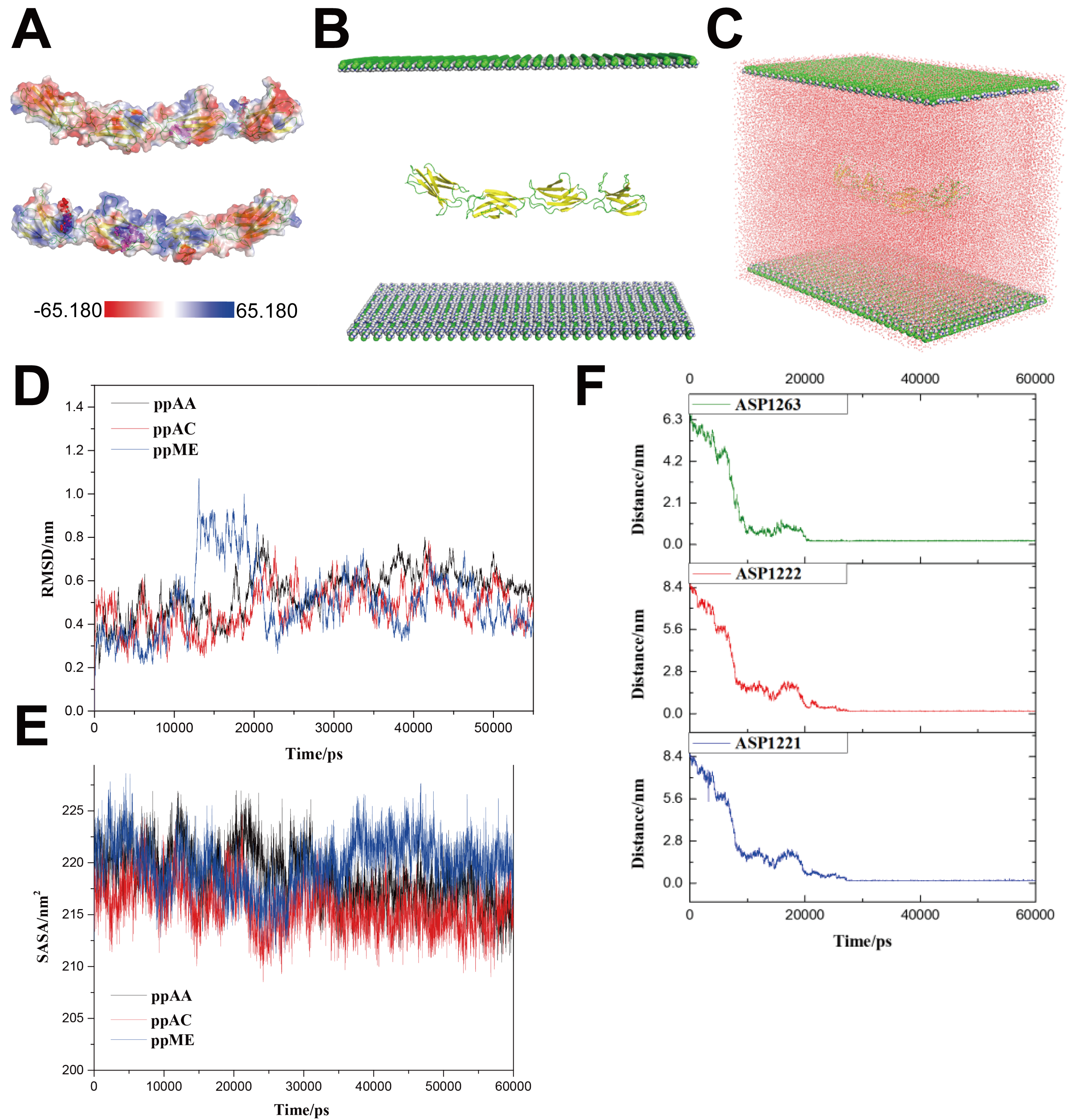

Supplement: Supplementary file 3 — Additional file 3: Figure S3. Establishment of the molecular dynamics simulation system. A) 3D diagram of the key fragment of the FN molecule, where the red and blue indicators show the positively and negatively charged areas, respectively. FN III7-10 molecule floating above the chemical surface in the environment absent of solvent (B) and with solvent (C); Root Mean Square Deviation (RMSD) (D) and Solvent Accessible Surface Area (SASA) analysis (E) of FN III7-10 on the three surfaces during molecular dynamic simulation process. F) The distance between the amino acid residues of FN that formed hydrogen bonds with ppAA surface, and their alterations over time. [file 40824_2022_323_MOESM3_ESM.tif]

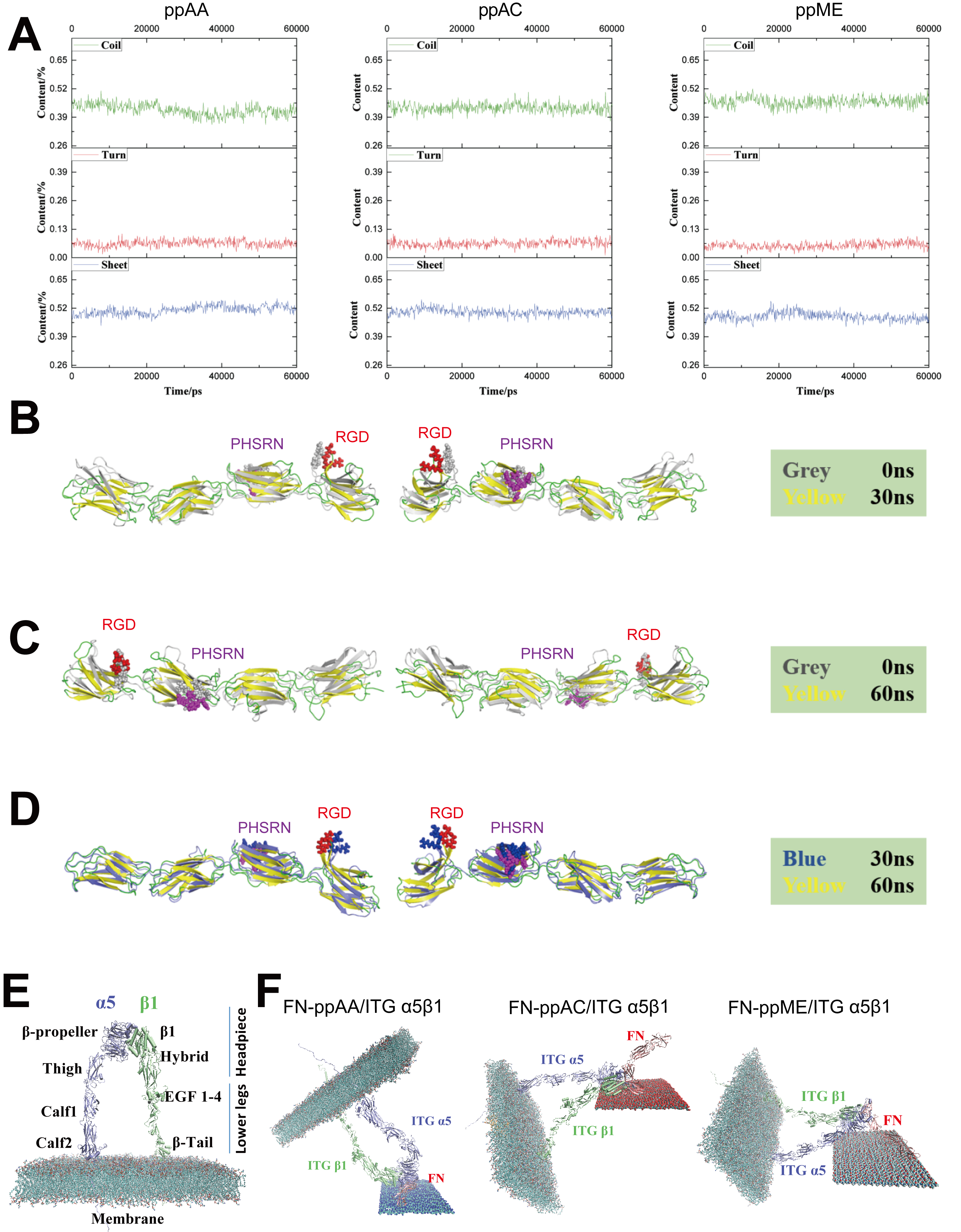

Supplement: Supplementary file 4 — Additional file 4: Figure S4. A) Fluctuation curve of the secondary structure of the FN molecule on the three chemical surfaces indicating the secondary structural alterations of the fibronectin during the molecular dynamics simulation; B-D) structural superposition analysis of FN in ppAA (B), ppAC (C), ppME (D) groups at different time points; E) the three-dimensional structure diagram of ITGα5β1 receptor protein in cell membrane; F) the optimal binding conformation of the FN-pp/ITGα5β1-membrane complex. [file 40824_2022_323_MOESM4_ESM.tif]

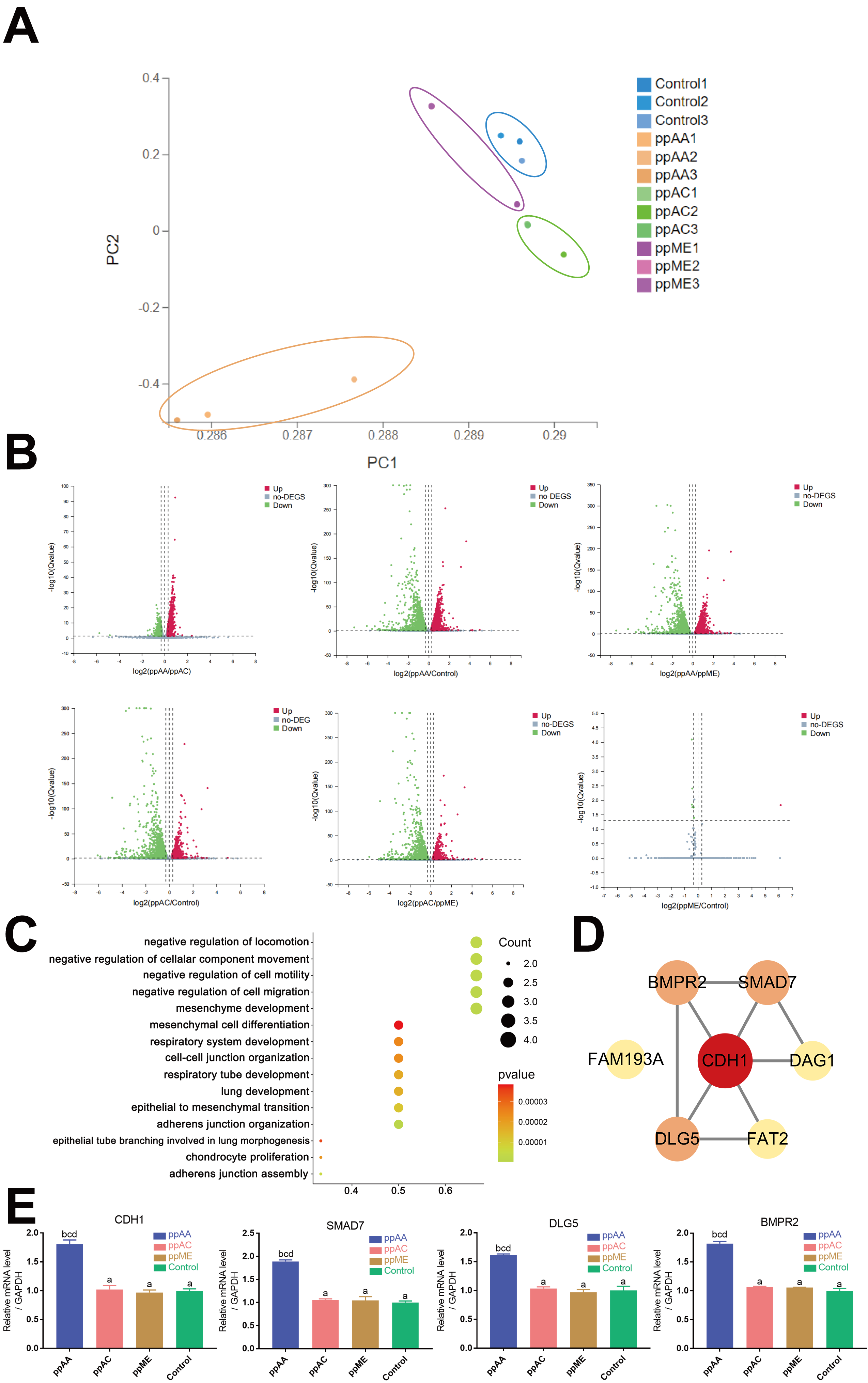

Supplement: Supplementary file 5 — Additional file 5: Figure S5. A) Principal component analysis of gene expression of HGEs on four surfaces; B) Volcano plot showed the differential expressed genes between the groups; C) Gene Ontology - Biological Process analysis of the co-upregulated cell-cell adherens junction related genes in ppAA group revealed a close relationship with the promotion of cell-cell junction organization; D) Protein-protein interaction anylysis of cell-cell adherens junction genes co-upregulated in ppAA compared with other groups. Dot size and color depth indicate connectivity in the network; E) The relative mRNA expression of CDH1, BMPR2, SMAD7, DLG5 genes in each group, detected via RT-qPCR assay. [file 40824_2022_323_MOESM5_ESM.tif]

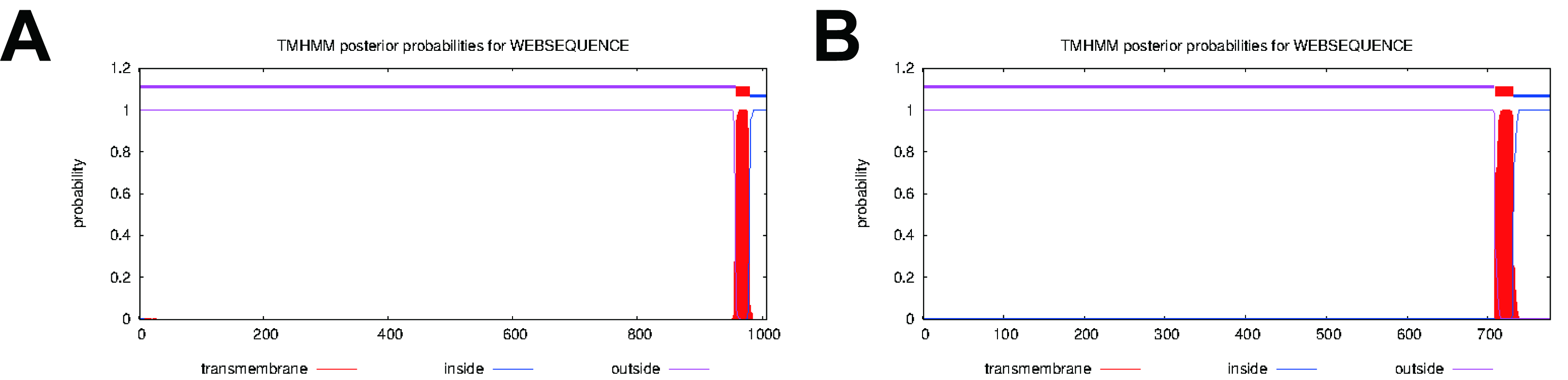

Supplement: Supplementary file 6 — Additional file 6: Figure S6. Prediction of the transmembrane structure of α5 chain (A) and β1 chain (B) of ITGα5β1 protein. [file 40824_2022_323_MOESM6_ESM.tif]
