# Supplementary material for: Plasma polymerized bio-interface directs fibronectin adsorption and functionalization to enhance “epithelial barrier structure” formation via FN-ITG β1-FAK-mTOR signaling cascade
Source: Biomater Res. 2022 Dec 26;26:88. doi: 10.1186/s40824-022-00323-0 (PMC9791785; doi:10.1186/s40824-022-00323-0)
Supplement: Supplementary file 7 — Additional file 7: Table S1. Deposition conditions for plasma polymerization of allylamine (AA), acrylic acid (AC) and methyl-oxazoline (ME). [file 40824_2022_323_MOESM7_ESM.docx]

**Table S1:** Deposition conditions for plasma polymerization of allylamine (AA), acrylic acid (AC) and methyl-oxazoline (ME).

|  | **Monomer** | **Pressure (mbar)** | **Power (W)** | **Time (s)** |
| --- | --- | --- | --- | --- |
| **ppAA** | Allylamine | 1.3 x 10^-1^ | 40 | 120 |
| **ppAC** | Acrylic acid | 1.3 x 10^-1^ | 10 | 120 |
| **ppME** | 2-methyl-oxazoline | 8.0 x 10^-2^ | 50 | 120 |
