# Supplementary material for: Plasma polymerized bio-interface directs fibronectin adsorption and functionalization to enhance “epithelial barrier structure” formation via FN-ITG β1-FAK-mTOR signaling cascade
Source: Biomater Res. 2022 Dec 26;26:88. doi: 10.1186/s40824-022-00323-0 (PMC9791785; doi:10.1186/s40824-022-00323-0)
Supplement: Supplementary file 8 — Additional file 8: Table S2. Primer used in the RT-qPCR assays. [file 40824_2022_323_MOESM8_ESM.docx]

**Table S2: Primer used in the RT-qPCR assays**

| **Genes** | **Primer sequences** |
| --- | --- |
| PDK1 | **Forward:** 5’- GGCTGGTTTTGGTTATGGATTG -3’ |
|  | **Reverse:** 5’- CTGGGAGTCTTTCTATTGAGTCTG -3’ |
| mTOR | **Forward:** 5’- CAAGAACTCGCTGATCCAAATG -3’ |
|  | **Reverse:** 5’- GCTGTACGTTCCTTCTCCTTC -3’ |
| RPS6K | **Forward:** 5'- ACCCCTTTATTGTGGAACTGG -3' |
|  | **Reverse:** 5'- GTAGAAGCAGGCCGTATCTTC -3' |
| PIK3CA | **Forward:** 5’- GAGTACCTTGTTCCAATCCCAG -3’ |
|  | **Reverse:** 5’- TTCCTCTTTAGCACCCTTTCG -3’ |
| PIK3CB | **Forward:** 5’- CTATCCAGACCAGTACGTTCG -3’ |
|  | **Reverse:** 5’- GAGGGCACAATCAAGAAAAGG -3’ |
| 4EBP | **Forward:** 5'- CAATATCCCAGGAGTCACTAGC -3' |
|  | **Reverse:** 5'- CTTGCAGGAGAGTCAGATGTC -3' |
| PTEN | **Forward:** 5'- AAGGGACGAACTGGTGTAATG -3' |
|  | **Reverse:** 5'- GCCTCTGACTGGGAATAGTTAC -3' |
| Eif4A | **Forward:** 5'- TTGTACTGGATGAAGCTGACG -3' |
|  | **Reverse:** 5'- TCACCTCAAGCACATCAGAAG -3' |
| Eif4B | **Forward:** 5’- AATTTGAGGACCTGGATTCCC -3’ |
|  | **Reverse:** 5’- TCTATCACGGCCAAAAGAACG -3’ |
| Eif4G | **Forward:** 5'- GGACCTAGATTAGACCATGAACG -3' |
|  | **Reverse:** 5'- GTTCTCGCAACTCTACGGTATC -3' |
| AKT1 | **Forward:** 5’- TCTATGGCGCTGAGATTGTG -3’ |
|  | **Reverse:** 5’- TCTTAATGTGCCCGTCCTTG -3’ |
| AKT2 | **Forward:** 5'- CGGTTTTATGGTGCAGAGATTG -3' |
|  | **Reverse:** 5'- AGTCAGTGATCTTGATGTGGC -3' |
| FAK | **Forward:** 5’- CTCCTACTGCCAACCTGGAC -3’ |
|  | **Reverse:** 5’- GCCGACTTCCTTCACCATAG -3’ |
| Plectin | **Forward:** 5’- ACAAGGCGGATAGCATGATC -3’ |
|  | **Reverse:** 5’- GTAGGTTGTACTCGGTGCG -3’ |
| ITG β1 | **Forward:** 5'- TGGAGGAAATGGTGTTTGC -3' |
|  | **Reverse:** 5'- CGTTGCTGGCTTCACAAGTA -3' |
| CDH1 | **Forward:** 5'- CTCGACACCCGATTCAAAGT -3' |
|  | **Reverse:** 5'- CCGTAGAGGCCTTTTGACTG -3' |
| SMAD7 | **Forward:** 5'- TTCCTCCGCTGAAACAGGG -3' |
|  | **Reverse:** 5'- CCTCCCAGTATGCCACCAC -3' |
| BMPR2 | **Forward:** 5'- GGCAGCAGTATACAGATAGGTG -3' |
|  | **Reverse:** 5'- CTGCCCTGTTACTGCCATTATT -3' |
| DLG5 | **Forward:** 5'- GCAGGTGTTGAAGCACAACG -3' |
|  | **Reverse:** 5'- CCGAAGGGCGTCATAGTCC -3' |
| GAPDH | **Forward:** 5'- TCAGCAATGCCTCCTGCAC -3' |
|  | **Reverse:** 5'- TCTGGGTGGCAGTGATGGC -3' |
